# Immunosenescence and bone remodeling: from immune cell senescence to skeletal aging through the bone marrow microenvironment

**DOI:** 10.3389/fimmu.2026.1979955

**Published:** 2026-10-20

**Authors:** Jinliang Zhuang, Weiwei Yu

**Affiliations:** 1 Department of Orthodontics, Hospital of Stomatology, Jilin University, Changchun, China; 2 Department of Oral Mucosal Diseases & Oral Emergency, Hospital of Stomatology, Jilin University, Changchun, China

**Keywords:** bone remodeling, immunosenescence, inflammaging, osteoimmunology, osteoporosis, senescence-associated secretory phenotype

## Abstract

With advancing age, the immune system undergoes progressive remodeling termed immunosenescence, which is intimately linked to skeletal homeostasis through the shared bone marrow microenvironment. This review systematically examines how senescent innate and adaptive immune cells disrupt bone remodeling by altering the cytokine milieu and releasing senescence-associated secretory phenotype (SASP) factors. Aged macrophages exhibit enhanced M1 proinflammatory polarization, while neutrophils display increased oxidative burst and delayed apoptosis. Natural killer cells lose cytotoxic capacity, and dendritic cells show impaired antigen presentation yet elevated basal inflammation. Within the adaptive compartment, T helper 17 (Th17) cells expand, regulatory T cells (Tregs) undergo functional decline and lineage instability, and B cells shift from osteoprotegerin (OPG) production toward receptor activator of nuclear factor kappa B ligand (RANKL) expression. These changes collectively elevate the RANKL/OPG ratio, promote osteoclastogenesis, and suppress osteoblast function. Systemic factors including estrogen deficiency and gut dysbiosis further amplify this immune-driven bone loss. Emerging therapeutic strategies range from conventional anti-resorptive agents to senolytics and immune modulators that restore marrow homeostasis. Understanding immunosenescence as an important contributor to skeletal aging provides a conceptual framework for immune-centered prevention and treatment of osteoporosis and fracture complications in the elderly, while recognizing that direct human causal evidence remains limited for several immune cell populations.

## 1 Introduction

Bone is a dynamically renewing organ throughout life. Through bone remodeling, bone tissue continuously removes old or damaged matrix and replaces it with newly formed bone, thereby preserving structural integrity, repairing microdamage, and maintaining calcium and phosphorus homeostasis (1). The basic functional unit of bone remodeling is the basic multicellular unit (BMU), which proceeds through five sequential phases: activation, resorption, reversal, formation, and quiescence. During the resorption phase, osteoclasts attach to the bone surface, create a sealed microenvironment, and dissolve mineralized matrix while degrading organic components via secretion of hydrochloric acid and cathepsin K. During the reversal phase, osteoclasts withdraw, mononuclear cells clean the resorption lacunae, and the surface is prepared for new bone deposition. During the formation phase, osteoblasts deposit osteoid within the resorption lacunae and promote its subsequent mineralization (2, 3). Under normal physiological conditions, bone resorption and formation are tightly coupled in both time and space; the volume of bone resorbed and formed within a single BMU is approximately equivalent, so that overall bone mass remains constant (4).

Osteoblasts are derived from bone marrow mesenchymal stem cells (MSCs) and mature through a differentiation program driven by the transcription factors Runx2 and Osterix. They secrete type I collagen and osteocalcin, and initiate mineralization via matrix vesicles (5). Upon completion of bone formation, osteoblasts undergo one of three fates: apoptosis, transformation into bone lining cells, or entombment within the mineralized matrix as osteocytes (6). During aging, both the number and functional capacity of osteoblasts decline, characterized by slowed proliferation, impaired mineralization, and increased apoptosis, leading to a relative insufficiency in bone formation (7, 8). Importantly, osteoblasts are also targets of immune signals; cytokine receptors expressed on their surface enable them to sense and respond to alterations in the bone marrow immune environment, which constitutes the cellular foundation for how immunosenescence influences bone formation (5).

It is essential to distinguish bone remodeling from bone modeling. In bone modeling, resorption and formation occur on separate bone surfaces and determine bone shape and dimensions; this process is predominantly active during growth and development. In contrast, bone remodeling involves sequential resorption and formation within the same BMU and represents the principal mode of adult bone renewal (1, 2). With advancing age, the fundamental characteristics of remodeling change: although the activation frequency may increase in old age, the amount of bone formed by each BMU decreases, resulting in trabecular thinning and discontinuity, increased cortical porosity, and an overall negative remodeling balance (8, 9). In postmenopausal women, the activation frequency of remodeling rises markedly, the resorption phase is prolonged while the formation phase is shortened, and the negative balance per unit bone volume is further amplified (10). A thorough understanding of these fundamental processes is a prerequisite for analyzing how immunosenescence disrupts bone remodeling.

The coupling between bone resorption and formation depends on multiple mechanisms. Osteoclasts and their precursors secrete coupling factors such as platelet-derived growth factor B-chain homodimer and sphingosine-1-phosphate, which recruit and stimulate osteoblast precursors. Transforming growth factor-β (TGF-β) and insulin-like growth factor 1, released from the bone matrix during resorption, also promote osteogenesis in a paracrine manner (9, 11). In recent years, the reversal phase has been recognized not merely as a transitional stage but as a regulatory window in which coupling signals are highly active; the density and functional status of reversal-phase cells directly influence the efficiency of subsequent bone formation (3). When coupling mechanisms become imbalanced, whether through enhanced resorption or impaired formation, net bone loss ensues, representing the common pathological basis of metabolic bone diseases such as osteoporosis (4).

Osteocytes are the most abundant and longest-lived cells in bone tissue. They arise from terminal differentiation of osteoblasts, are embedded within the mineralized matrix, and sense mechanical loading and microdamage through an extensive dendritic network. Osteocytes serve not only as mechanosensors of bone remodeling but also as key endocrine and paracrine cells: their secretion of sclerostin inhibits Wnt signaling and thereby negatively regulates bone formation, whereas their expression of receptor activator of nuclear factor-κB ligand (RANKL) constitutes the primary source of RANKL required for osteoclast generation during physiological bone remodeling (6). Lineage tracing and conditional gene knockout studies have confirmed that, during adult bone remodeling, the RANKL required for osteoclast generation is provided mainly by osteocytes rather than by osteoblasts or bone lining cells, a finding that places osteocytes at the core of bone resorption regulation (12).

## 2 Osteoimmunology fundamentals

### 2.1 RANKL/RANK/OPG axis

The RANKL/RANK/osteoprotegerin (OPG) axis is the central regulatory pathway governing osteoclast differentiation and activation, and also serves as the molecular hub connecting the immune and skeletal systems. Between 1997 and 1998, multiple research groups independently identified this system: OPG functions as a soluble decoy receptor of the tumor necrosis factor receptor superfamily, capable of binding and neutralizing RANKL. Upon binding of RANKL (also known as osteoclast differentiation factor) to RANK on the surface of osteoclast precursors, it drives their differentiation into mature osteoclasts (13–15). OPG knockout mice develop early-onset severe osteoporosis due to uncontrolled osteoclastogenesis, whereas OPG overexpression results in osteopetrosis, genetically establishing the fundamental principle that the RANKL/OPG ratio determines the intensity of bone resorption (15, 16).

Following RANKL binding to RANK, downstream nuclear factor-κB (NF-κB), mitogen-activated protein kinase (MAPK) (including p38, c-Jun N-terminal kinase, and extracellular signal-regulated kinase), and calcineurin signals are activated via the adaptor protein TRAF6, ultimately inducing and activating the transcription factor nuclear factor of activated T cells cytoplasmic 1 (NFATc1). NFATc1 is the master regulator of osteoclast terminal differentiation and can directly initiate transcription of osteoclast signature genes, including tartrate-resistant acid phosphatase, cathepsin K, and the calcitonin receptor (17). Under the synergistic survival and proliferation signals provided by macrophage colony-stimulating factor, RANKL signaling is sufficient to drive mononuclear macrophage lineage precursors through the entire differentiation program to become multinucleated mature osteoclasts (16). The discovery of this regulatory axis is regarded as a milestone in bone biology and laid the foundation for the subsequent development of anti-RANKL therapeutics (18, 19). The expression of the RANKL/RANK/OPG system is not restricted to bone tissue; it also plays important roles in lymph node development, mammary gland formation, thermoregulation, and immune cell function. This broad distribution pattern itself suggests that the skeletal and immune systems share regulatory modules from an evolutionary standpoint (20, 21).

The physiological significance of the RANKL/RANK/OPG system extends beyond osteoclast differentiation per se. RANKL-RANK signaling participates in lymph node organogenesis, thymic medullary epithelial cell differentiation, mammary lobule formation, and thermoregulatory center modulation, while OPG inhibits abnormal calcification in the vascular system (20, 21). The discovery of this system transcended the traditional disciplinary boundaries between bone biology and immunology; its very nomenclature reflects this interdisciplinary nature: RANKL was initially identified as a T cell-dendritic cell (DC) interaction molecule RANKL, and was subsequently independently characterized as osteoclast differentiation factor (18, 19). From a regulatory perspective, RANKL expression is subject to multifactorial control by hormones (such as estrogen, vitamin D), mechanical loading, and inflammatory factors, making it a molecular node where systemic signals converge to regulate bone resorption (16). In the context of aging, the upregulation of RANKL expression in osteocytes and immune cells, together with insufficient compensatory OPG secretion, jointly leads to axis imbalance (12, 22).

### 2.2 Emergence of osteoimmunology

Osteoimmunology, as an interdisciplinary field, aims to investigate the shared cells, molecules, and regulatory mechanisms between the immune and skeletal systems. The origin of this field can be traced to the discovery that activated T cells express RANKL and directly regulate osteoclast generation: in 1999, Kong et al. demonstrated that activated T lymphocytes participate in bone loss and joint destruction in adjuvant arthritis by expressing the OPG ligand (i.e., RANKL), establishing adaptive immune cells for the first time as direct drivers of osteoclastogenesis (23). In 2007, Takayanagi systematically elaborated the evidence that the immune and skeletal systems share RANKL, NFATc1, costimulatory molecules, and multiple cytokines, formally proposing the conceptual framework of osteoimmunology (24). Over the subsequent two decades, research has demonstrated that immune cells not only promote bone resorption by secreting RANKL and inflammatory factors, but also inhibit osteoclast generation through interferon-γ (IFN-γ), interleukin (IL)-10, and TGF-β, and additionally influence bone formation by modulating osteoblast and osteocyte function (25, 26).

The deepening of osteoimmunology research has brought about an important shift in the understanding of age-related bone loss. The traditional view attributes senile osteoporosis primarily to osteoblast functional decline and sex hormone deficiency, whereas the osteoimmunology perspective emphasizes that bone marrow hematopoiesis and bone remodeling share the same microenvironment; therefore, alterations in the cytokine network secreted by immune cells necessarily affect bone metabolism (27). The systemic remodeling of the immune system that occurs during aging, known as immunosenescence and intertwined with chronic low-grade inflammation, is increasingly recognized as an important contributor to bone remodeling imbalance (28, 29). This review centers on the theme of immune cell senescence, systematically summarizing the phenotypes and mechanisms of innate and adaptive immune cell aging, analyzing the effects of senescent immune cells on osteoblasts, osteoclasts, and bone remodeling through the senescence-associated secretory phenotype (SASP) and cytokine networks, and discussing intervention strategies targeting immunosenescence. To integrate these multi-level interactions, we first present a conceptual framework illustrating how senescent immune cells collectively disrupt bone remodeling homeostasis (**Figure 1**).

**Figure 1:**
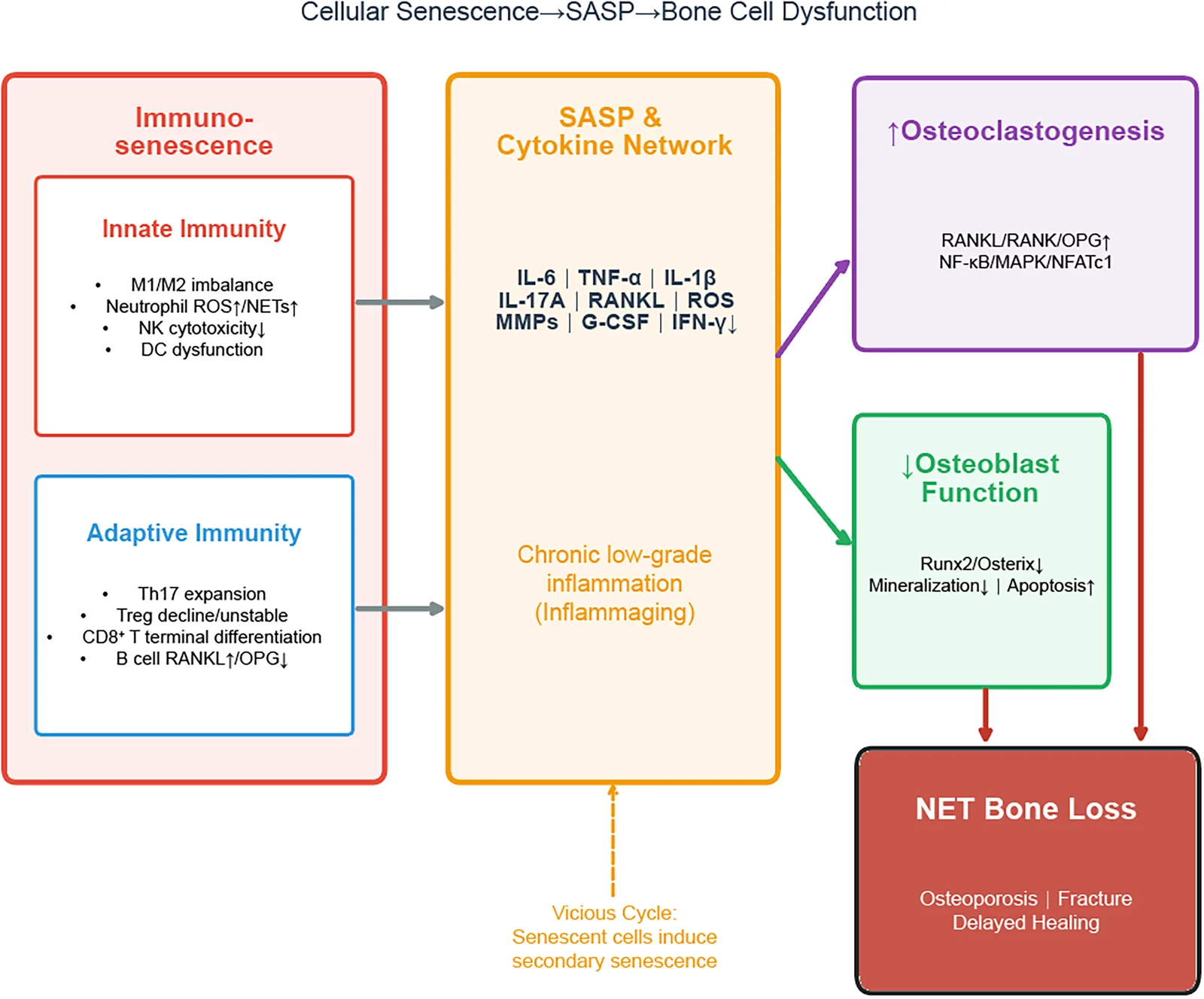
Immunosenescence-driven bone remodeling imbalance. Senescent innate and adaptive immune cells within the bone marrow microenvironment release SASP factors including IL-6, TNF-α, IL-1β, IL-17A, and RANKL. These mediators collectively enhance osteoclastogenesis via NF-κB/MAPK/NFATc1 signaling while suppressing osteoblast differentiation and mineralization through Runx2 inhibition and increased apoptosis, ultimately resulting in net bone loss and skeletal fragility. ↓ indicates decrease, ↑ indicates increase. Adobe Illustrator 2026 software was used to generate the images.

Several recent reviews have covered the intersection of osteoimmunology, immunosenescence, inflammaging, and age-related bone loss, and the present review builds on this foundation while differing from it in three respects. First, whereas previous reviews generally treat immune aging as a systemic background factor, we focus specifically on the bone marrow microenvironment as the shared niche in which immune senescence and skeletal aging proceed as an interconnected process. Second, we highlight genuinely emerging evidence, including bone marrow-specific immune senescence revealed by single-cell and spatial omics (Section 3.6) and direct causal evidence linking senescent immune subsets to skeletal phenotypes, rather than restating well-established associations. Third, we evaluate the strength of causal evidence separately for each immune-cell population and distinguish findings obtained in physiological aging from findings extrapolated from inflammatory disease models. On this basis, the therapeutic section stratifies interventions by their level of clinical readiness, from approved osteoporosis drugs to investigational senolytic and immune-modulatory strategies.

## 3 Biology of immunosenescence

### 3.1 Concept and features

Immunosenescence refers to the progressive structural and functional degeneration of the immune system with advancing age. Its overall consequences include diminished responses to novel antigens, weakened vaccine efficacy, increased susceptibility to infections and malignancies, and concomitant abnormal inflammatory reactions directed against self-antigens and harmless environmental antigens (30, 31). Immunosenescence is not a change confined to a single cell population; rather, it involves systemic remodeling of central immune organs such as the thymus and bone marrow, as well as various peripheral immune cell subsets (32). At the innate immunity level, aging neutrophils exhibit impaired chemotaxis and phagocytosis, Toll-like receptor signaling and efferocytosis in mononuclear macrophages are compromised, and the cytotoxicity of natural killer (NK) cells declines. At the adaptive immunity level, naive T and B cell pools atrophy, memory cell proportions increase, T cell receptor and B cell receptor diversity decreases, and terminally differentiated senescent phenotypes accumulate in substantial numbers (33, 34).

Throughout this review we use the following terms in a restricted and consistent sense. Cellular senescence denotes stable cell cycle arrest accompanied by a senescence-associated secretory phenotype and supported by marker combinations such as p16INK4a or p21CIP1 expression together with senescence-associated beta-galactosidase activity; no single marker is sufficient for firm identification. Immunosenescence denotes the broader age-associated remodeling of the immune system, including changes in subset composition, receptor repertoire, and effector function that do not necessarily reflect cellular senescence. We use aged immune cells as a neutral term for cells isolated from older individuals, and we reserve senescent immune cells for populations in which senescence markers or equivalent experimental evidence have been demonstrated. Age-associated functional alterations, including impaired chemotaxis, altered polarization, increased basal inflammatory secretion, reduced cytotoxicity, and changes in cell frequency, are discussed as functional dysregulation unless senescence markers are available. For short-lived and highly dynamic populations such as neutrophils, whose circulating lifespan is measured in hours, true cellular senescence is difficult to establish, and their age-associated phenotypes are therefore interpreted as aged or dysfunctional states rather than bona fide senescence. Exhaustion refers to a distinct differentiation state driven by sustained antigen receptor stimulation and checkpoint expression, and it is not used interchangeably with senescence.

From a systemic perspective, immunosenescence is both a consequence of organismal aging and a driver of multi-organ aging. Senescent immune cells promote degenerative changes in distant organs, including blood vessels, nerves, muscles, and bone, through the secretion of inflammatory mediators and SASP factors (32). The development of single-cell omics technologies has enabled researchers to map human immunosenescence at single-cell resolution, identifying senescence-associated T cell, B cell, and myeloid cell subsets that expand with age, and revealing significant interindividual differences in the rate of immunosenescence. This suggests that immune age may predict the risk of aging-related diseases more accurately than chronological age (35, 36).

Clinical evaluation of immunosenescence relies on immune cell subset analysis, functional assays, and serum inflammatory marker detection. The classical immune risk phenotype concept centers on CD4/CD8 ratio inversion, cytomegalovirus (CMV) seropositivity, and expansion of specific T cell subsets as core indicators, and these parameters correlate with mortality in elderly individuals (37). Recent multi-omics studies have further proposed an immune age score that integrates transcriptomic, proteomic, and cellular composition data to quantify individual immunosenescence (32, 35). The rate of immunosenescence exhibits considerable individual heterogeneity; genetic background, chronic infection history, nutrition, exercise, and metabolic status all influence its progression (31). This heterogeneity suggests that interventions targeting immunosenescence require stratified strategies, and also provides an immunological perspective for explaining differences in osteoporosis risk among elderly populations.

### 3.2 Inflammaging

Inflammaging, proposed by Franceschi et al., refers to the systemic, persistent, low-grade inflammatory state that develops in aging individuals without overt infection. It is characterized by elevated serum levels of inflammatory markers such as IL-6, tumor necrosis factor-α (TNF-α), and C-reactive protein (38, 39). The sources of inflammaging are multifaceted: senescent cells accumulate and release SASP factors; intestinal barrier function declines, allowing microbial products to translocate into the bloodstream; mitochondrial dysfunction releases damage-associated molecular patterns; obesity and adipose tissue inflammation amplify systemic cytokine levels; and the diminished ability of the immune system to clear senescent cells and pathogens further prolongs inflammatory stimulation (40, 41). Inflammaging is now recognized as one of the hallmarks of organismal aging and has been causally linked to multiple aging-related diseases, including atherosclerosis, neurodegenerative diseases, type 2 diabetes, and osteoporosis (42, 43).

Inflammaging and immunosenescence are mutually causal and reinforcing. On one hand, the chronic inflammatory environment promotes immune cell terminal differentiation and exhaustion; on the other hand, senescent immune cells themselves constitute an important source of inflammatory factors and propagate the senescent phenotype to neighboring cells through SASP (44, 45). Intervention studies targeting inflammaging suggest that inhibiting key signaling nodes such as NF-κB, the NOD-like receptor family pyrin domain containing 3 (NLRP3) inflammasome, and mechanistic target of rapamycin (mTOR), or lifestyle interventions such as caloric restriction and exercise, can alleviate low-grade inflammation and delay immune functional degeneration to a certain extent (46). This theoretical framework is particularly important for understanding age-related bone loss: bone marrow is an organ densely populated with immune cells, and the cytokine profile alterations caused by inflammaging directly act on osteogenic and osteoclastic lineages, constituting the macroscopic background for how immunosenescence affects bone remodeling (28).

Bone marrow is one of the organs where the effects of inflammaging are most concentrated. Bone marrow hematopoiesis shifts toward myeloid lineages with age; hematopoietic stem cell numbers increase while lymphoid output decreases, bone marrow adipocytes accumulate, and the cytokine profile of the stromal microenvironment tilts toward a proinflammatory direction (41, 47). The elevated levels of IL-6, IL-1β, TNF-α, and chemokines in the local bone marrow originate from both senescent hematopoietic and stromal cells and accumulated senescent immune cells (28). The receptors for these factors are widely expressed on osteogenic lineage cells and osteoclast precursors, enabling bone marrow inflammaging to be directly translated into bone remodeling signals (7). Therefore, age-related bone loss can be understood as the local expression of systemic inflammaging in the skeletal organ.

### 3.3 Thymic involution and T cell remodeling

The thymus is the central organ for T cell development and also the organ that degenerates earliest and most prominently during immunosenescence. In humans, the thymus begins to undergo fatty infiltration after puberty, and functional thymic tissue gradually diminishes, leading to a continuous decline in naive T cell output (48). The direct consequence of thymic involution is that renewal of the peripheral T cell pool relies mainly on homeostatic proliferation of existing cells rather than thymopoiesis, resulting in narrowed T cell receptor diversity, decreased proportions of naive cells, and increased proportions of memory and effector cells (49). Animal studies have shown that inducing premature thymic involution in young mice can replicate peripheral T cell phenotypes similar to natural aging, including reduced naive cells and accumulation of senescence-associated T cells, proving that thymic involution is an upstream driver of T cell aging rather than a mere concomitant phenomenon (49). Correspondingly, enhancing thymic function in aged mice can improve T cell reconstitution and restore partial immune responsiveness, suggesting that the thymus is a potential intervention target for delaying immunosenescence (50). Thymic involution also weakens the maintenance of central immune tolerance, allowing autoreactive T cells to escape more readily into the periphery, laying the groundwork for chronic inflammation and autoimmune tendencies. This mechanism has potential connections with the enhancement of autoreactive inflammation in aged bone marrow (32).

The indirect effects of thymic involution on bone metabolism deserve attention. Reduced thymic output shifts the maintenance of the peripheral T cell pool toward homeostatic proliferation, a process accompanied by compensatory changes in homeostatic cytokines such as IL-7 and IL-15, which are important regulators of T cell-mediated bone loss (51, 52). The reduction in thymus-derived regulatory T cell (Treg) output also weakens the renewal of peripheral immune tolerance, compounding the functional decline of aged Tregs (48). Interventions that enhance thymic function, such as growth hormone, IL-7, or thymic regeneration strategies, merit evaluation for their potential effects on bone metabolism while improving immune reconstitution (50).

### 3.4 Chronic viral infections

Persistent viral infections such as CMV are important exogenous drivers of adaptive immunosenescence. CMV infection rate increases with age in the population. Following latent infection, the virus undergoes repeated intermittent reactivation, continuously stimulating clonal expansion of CD8^+^ T cells and forming the characteristic phenomenon of memory inflation (37). CMV-seropositive elderly individuals show significantly elevated proportions of highly differentiated CD28^−^ CD8^+^ T cells in peripheral blood, reduced T cell receptor repertoire diversity, and associations with weakened vaccine responses and increased all-cause mortality (53). Recent studies have also identified bone marrow as an important reservoir for highly differentiated CD8^+^ effector memory T cells; CMV infection increases local IL-15 production in bone marrow, providing niche support for the long-term survival of these cells (52). This phenomenon suggests that bone marrow is not only the site of bone remodeling but also a sanctuary for the residence and expansion of senescent immune cells. The enrichment of senescent T cells in the local bone marrow allows the inflammatory mediators they secrete to act directly on bone cell lineages. At the molecular level, MAPK-activated protein kinase 2 and other signaling molecules have been shown to participate in regulating CMV-driven memory T cell inflation, providing clues for intervening in virus-related immunosenescence (54).

The relationship between persistent viral infection and bone metabolism also has direct evidence. Decreased bone density in patients with chronic inflammatory viral infections has been repeatedly reported, with mechanisms involving virus-associated immune activation and T cell subset alterations (37, 55). CMV-driven T cell subset remodeling correlates with systemic inflammation levels in elderly individuals, indirectly affecting the bone remodeling environment (53). These observations suggest that controlling chronic viral infections or mitigating their immune consequences may become an adjunct strategy for maintaining skeletal health in old age (32).

### 3.5 Molecular and metabolic mechanisms

At the molecular level, immunosenescence manifests as the superposition of multiple mechanisms, including telomere shortening, DNA damage accumulation, epigenetic drift, proteostasis imbalance, and mitochondrial dysfunction (34, 44). Epigenetic studies have identified conserved chromatin remodeling patterns during T cell aging, including altered accessibility of regulatory regions associated with cell cycle arrest and inflammatory transcription programs. These epigenetic imprints are highly conserved across species and pathological contexts, suggesting the existence of programmed senescence regulatory axes (56). Metabolic reprogramming is another core dimension of immunosenescence: senescent immune cells exhibit imbalance between glycolysis and oxidative phosphorylation, decreased mitochondrial respiratory function, and increased reactive oxygen species (ROS) generation. The dysregulation of mTOR and AMPK pathways further affects the differentiation direction and effector functions of immune cells (57). The phenotype and function of memory T cells in bone marrow undergo characteristic changes with age, including altered expression of homing receptors and cytokine secretion profiles, causing the cytokine composition of the bone marrow microenvironment to tilt with aging (51). These molecular and metabolic changes collectively constitute the intrinsic basis for the functional abnormalities of senescent immune cells and also provide a mechanistic framework for understanding their paracrine effects.

At the signaling pathway level, persistent NF-κB activation is a shared molecular feature of both immunosenescence and inflammaging, driving continuous low-level transcription of inflammatory genes (46). mTOR signaling becomes dysregulated with age, affecting immune cell proliferation, differentiation, and metabolic adaptation; the mTOR inhibitor rapamycin delays aging-related phenotypes in multiple models and is one of the representative strategies for intervening in immunosenescence (44, 46). Age-related activation of the NLRP3 inflammasome translates metabolic stress into the mature release of IL-1β and IL-18, directly contributing to the cytokine profile of inflammaging (41). These pathways also play important roles in osteoclast generation and osteoblast function regulation, constituting molecular convergence points for the effects of immunosenescence on bone metabolism (42).

The feasibility of intervention in immunosenescence has been preliminarily validated at multiple levels. In addition to lifestyle interventions such as caloric restriction and exercise, pharmacological strategies include mTOR inhibition, senescent cell clearance, thymic regeneration, and metabolic modulation (34, 46). The potential effects of these interventions on bone metabolism are beginning to receive attention: improving the state of immunosenescence could theoretically simultaneously alleviate the pressure of inflammaging on bone remodeling (32). However, data on bone metabolic outcomes from immune interventions in elderly populations remain limited, with most bone metabolism evidence coming from indirect observations (43). Incorporating bone density, bone turnover markers, and fracture risk as endpoint indicators in immunosenescence intervention trials is an urgently needed step in this interdisciplinary field (28).

### 3.6 Single-cell and spatial omics of bone marrow immune aging

Single-cell and spatial omics technologies have begun to resolve how immune aging unfolds within the bone marrow niche itself, rather than requiring inference from peripheral blood. Single-cell transcriptomic studies of human aging have identified senescence-associated T cell, B cell, and myeloid subsets that expand with age, and they have shown that the rate of immune aging varies considerably between individuals, supporting an immune age concept that may predict aging-related disease risk more accurately than chronological age (35, 36). A consensus single-cell reference atlas of healthy human bone marrow spanning a wide age range has revealed an age-correlated decline of lymphoid-bipened progenitors consistent with the myeloid skew of aging hematopoiesis, a pronounced age-associated shrinkage of naïve CD8^+^ T cells, expansion of effector and memory CD4^+^ T cells, and cell type-specific aging-related transcriptional programs (58). In the bone marrow of cytomegalovirus-seropositive individuals, highly differentiated CD8^+^ effector and memory T cells accumulate in response to increased local IL-15 production, illustrating how a persistent viral driver can establish a bone marrow reservoir of proinflammatory T cells (52).

These immune changes proceed concurrently with remodeling of the skeletal and stromal compartments, and spatial omics has begun to define where these interactions physically occur. With aging, bone marrow adiposity increases, stromal cells acquire a proinflammatory secretory profile, and niche support for skeletal stem and progenitor cells declines (47, 59). Spatially resolved multiomic mapping of human bone marrow has demonstrated that early myeloid progenitors localize to a relatively hyperoxygenated arterio-endosteal neighborhood whereas hematopoietic stem and progenitor cells associate closely with adipocytes, revealing that niche topology, rather than cell composition alone, instructs local hematopoietic and immune behavior (60). Combined single-cell and spatial transcriptomics in mouse bone marrow have likewise resolved molecularly distinct Cxcl12-abundant reticular cell subsets that differentially localize to sinusoidal and arteriolar surfaces and act as local cytokine-secreting hubs (61).

Single-cell analyses have also localized cellular senescence to defined skeletal cell states within the marrow. Senescent osteocyte subpopulations with characteristic transcriptional signatures directly promote local bone destruction through pro-resorptive factor secretion (62), and osteocyte-specific senescence is sufficient to drive senescence of the surrounding bone and bone marrow (63). A cross-tissue transcriptomic senescence gene set has improved the identification of senescent cells and revealed senescence-associated pathways shared across organs (64), providing analytical tools with which senescent immune subsets in aged bone marrow can now be resolved.

Despite this progress, studies that simultaneously profile immune cells and skeletal cells in the aged human bone marrow at single-cell and spatial resolution remain scarce, and most current inferences derive from mouse models, peripheral blood, or non-spatial bone marrow analyses (35, 47). The spatial choreography of immune-skeletal interactions in physiological skeletal aging is therefore still largely uncharted. Bone marrow-focused single-cell and spatial atlases of aging are needed to define bone microenvironment-specific senescent immune subsets, to establish their physical proximity to osteogenic and osteoclastic lineages, and to guide the precision intervention strategies discussed in Section 10.

## 4 Cellular senescence and SASP in bone marrow

### 4.1 Cellular senescence and SASP

Cellular senescence is a stable cell cycle arrest state induced by telomere shortening, oncogene activation, oxidative stress, irradiation, or chemotherapy. It is accompanied by enlarged morphology, enhanced lysosomal activity (manifested as positive senescence-associated β-galactosidase), upregulated expression of cyclin-dependent kinase inhibitors such as p16^INK4a^ and p21^CIP1^, and extensive chromatin remodeling (64, 65). Although senescent cells exit the cell cycle, they maintain active metabolic and secretory functions. The various cytokines, chemokines, growth factors, proteases, and extracellular vesicles they secrete are collectively termed SASP (66, 67). The composition of SASP varies by cell type and inducing factor, but typically includes IL-6, IL-1β, IL-8, TNF-related molecules, monocyte chemoattractant proteins, matrix metalloproteinases (MMPs), and granulocyte-macrophage colony-stimulating factor (66). Mitochondrial dysfunction can induce SASP with unique compositional characteristics; the secretion profile of such senescent cells contains more prominent proinflammatory and pro-oxidative components (68). SASP has dual effects: under physiological conditions, chemokines in SASP recruit immune cells to clear senescent cells and participate in tissue repair and embryonic development; under pathological conditions, senescent cells continuously accumulate, SASP acts chronically on surrounding tissues, creates a chronic inflammatory environment, and induces secondary senescence in neighboring cells through paracrine mechanisms, causing the senescent phenotype to spread within tissues (67, 69).

The identification of senescent cells depends on a combination of multiple markers; a single marker is insufficient to define the senescent state. Transcriptomic studies have proposed cross-tissue senescent cell gene sets, improved the accuracy of senescent cell identification and predicting organ-commonality of senescence-related pathways (64). p16^INK4a^ and p21^CIP1^ represent the p16-Rb and p53-p21 senescence induction pathways, respectively; the timing and contribution of their activation vary by inducing factor (65). SASP secretion is regulated by transcriptional and signaling networks including NF-κB, CCAAT/enhancer-binding protein beta, mTOR, and GATA-binding protein 4. Post-translational modifications of SASP factors and extracellular vesicle sorting further increase its complexity (67). These molecular details provide a target basis for selectively intervening in SASP without disrupting the physiological functions of senescent cells (66).

The clearance of senescent cells *in vivo* depends on the combined surveillance of innate and adaptive immunity. NK cells and cytotoxic T cells recognize stress ligands and abnormal antigens on the surface of senescent cells and mediate their killing, while macrophages clear senescent cell debris through efferocytosis (69). Immunosenescence renders this surveillance system ineffective; senescent cells continuously accumulate in tissues due to clearance failure, forming a vicious cycle of increasing senescent cells and weakened immune surveillance. The classical INK-ATTAC transgenic mouse experiment demonstrated that drug-induced clearance of p16^INK4a^-positive senescent cells can delay the onset of multiple aging-related phenotypes, establishing for the first time *in vivo* a causal relationship between senescent cell accumulation and organismal aging phenotypes (70).

### 4.2 Senescence in bone cells

Bone tissue is a representative model for studying the relationship between cellular senescence and organ aging. Farr et al. first systematically identified the presence of senescent cells in the bone microenvironment, finding that p16^INK4a^ expression increases in bone tissue of aged mice, with senescent cells distributed among osteocytes, osteogenic lineage cells, and bone marrow stromal cells (71). Subsequent key studies employed both senescent cell clearance and p16^INK4a^-positive cell ablation strategies to demonstrate that targeting cellular senescence can prevent age-related bone loss in mice: after senescent cell clearance, trabecular and cortical bone mass and bone strength were improved, bone formation rate rebounded, and bone resorption declined (72). This series of work advanced bone aging research from correlative description to causal verification and also established the theoretical foundation for treating osteoporosis by clearing senescent cells (73).

Among various bone cells, osteocyte senescence has received the most attention. Osteocytes have a lifespan of decades and are deeply embedded in mineralized matrix, making them difficult to replace; they are therefore the bone cell type most prone to senescent cell accumulation (74). Senescent osteocytes highly express p16^INK4a^, p21^CIP1^, and multiple SASP factors, and alterations in their secretion profile can affect neighboring osteoblasts, osteoclast precursors, and bone marrow immune cells. Osteocyte-specific senescence models show that osteocyte senescence is sufficient to drive the overall senescent phenotype of bone and bone marrow, including bone mass loss and alterations in bone marrow hematopoietic composition, suggesting that osteocytes may be the initiating cells of bone tissue senescence (63). Single-cell transcriptomic analysis has further identified senescent osteocyte subpopulations with characteristic transcriptional features; these cells directly promote local bone destruction through the secretion of pro-resorptive factors (62). Epigenetic dysregulation is considered an important driver of osteocyte senescence; DNA methylation drift and chromatin accessibility changes can simultaneously affect osteocyte homeostatic maintenance and overall musculoskeletal system function (75). Senescent osteocytes upregulate RANKL expression and downregulate OPG, increasing the RANKL/OPG ratio, a change that constitutes one of the cellular bases for enhanced bone resorption in old age (8, 74).

The inducers of osteocyte senescence include oxidative stress, DNA damage, alterations in the mechanical microenvironment, and systemic SASP burden (74). The SASP of senescent osteocytes acts on the neighboring osteocyte network and can propagate senescence signals along the osteocytic canalicular system (63). Osteocytes also influence immune cell composition by regulating bone marrow hematopoiesis; the diminished support of senescent osteocytes for hematopoietic stem and progenitor cells indirectly participates in the process of bone marrow immunosenescence (47, 63). These findings make osteocytes a key cell type at the intersection of bone tissue senescence and immunosenescence (76).

### 4.3 Senescence of marrow stem/progenitor cells

The continuous replenishment of osteoblasts depends on the proliferation and osteogenic differentiation of bone marrow MSCs and skeletal stem progenitor cells. During aging, the number, self-renewal capacity, and differentiation fate of these stem progenitor cells all change: osteogenic differentiation weakens while adipogenic differentiation strengthens, leading to bone marrow fat accumulation and insufficient bone formation (7, 59). Single-cell sequencing studies have mapped the heterogeneity of skeletal stem progenitor cells in different bone compartments and their senescence trajectories, finding that stem progenitor cells in different locations have differential susceptibility to senescence. Long bone and craniofacial bone stem progenitor cells display different senescence characteristics (77). At the molecular level, upregulation of the chromatin remodeling factor NAP1L2 can drive MSC senescence and inhibit their osteogenic differentiation (78); abnormalities in the splicing factor YBX1 determine the osteogenic versus adipogenic fate choice of bone marrow stromal cells during aging through altered mRNA splicing patterns (79). The fate determination of osteogenic lineage cells is under the precise control of signaling pathways such as Wnt, BMP, and Hedgehog, as well as transcription factors such as Runx2 and Osterix; the disruption of these signaling and transcriptional networks by aging directly impairs bone formation capacity (5).

The interaction between skeletal cells and hematopoietic cells in the bone marrow microenvironment undergoes profound changes with aging. Bone marrow stromal cells provide niche signals for hematopoietic stem cells, while hematopoietic lineage cells in turn secrete cytokines that act on skeletal cells. The imbalance of this bidirectional dialogue is an important feature of aged bone marrow microenvironment degeneration (47). Notably, factors such as cardiotrophin-like cytokine factor 1 induced by exercise can partially reverse the senescent phenotypes of bone and muscle, suggesting that skeletal stem progenitor cell senescence is not irreversible (7). The above evidence collectively indicates that age-related bone formation decline is not only the result of intrinsic senescence of osteogenic lineage cells but also reflects the systemic changes in the overall bone marrow microenvironment, including immune cell composition.

### 4.4 SASP in skeletal aging

The SASP of the skeletal system (termed bone SASP by some scholars) has compositional features distinct from other organs. The factor spectrum secreted by senescent osteocytes and bone marrow cells prominently includes RANKL, IL-6, IL-1β, TNF-α, MMPs, and various chemokines, which are precisely the key mediators regulating osteoclast generation and osteoblast function (76). Bone SASP affects bone remodeling through three pathways: first, directly increasing the RANKL/OPG ratio and synergizing with inflammatory factors to promote osteoclast differentiation; second, inhibiting the osteogenic differentiation of MSCs and inducing osteoblast apoptosis; third, inducing secondary senescence in neighboring bone-forming cells through paracrine mechanisms, causing the senescent phenotype to spread within bone tissue (74, 76). Animal experiments show that local senescent cell clearance can only partially replicate the benefits of systemic clearance, indicating that bone tissue senescence is regulated by both local senescent cells and systemic senescent environments (80). This finding suggests that the systemic SASP burden contributed by immune cell senescence in bone marrow may be an important yet relatively underestimated factor affecting bone remodeling, and is the focus of subsequent chapters in this review. **Figure 2** illustrates the paracrine propagation mechanisms through which senescent immune cells transmit senescence signals to diverse bone cell lineages within the marrow niche.

**Figure 2:**
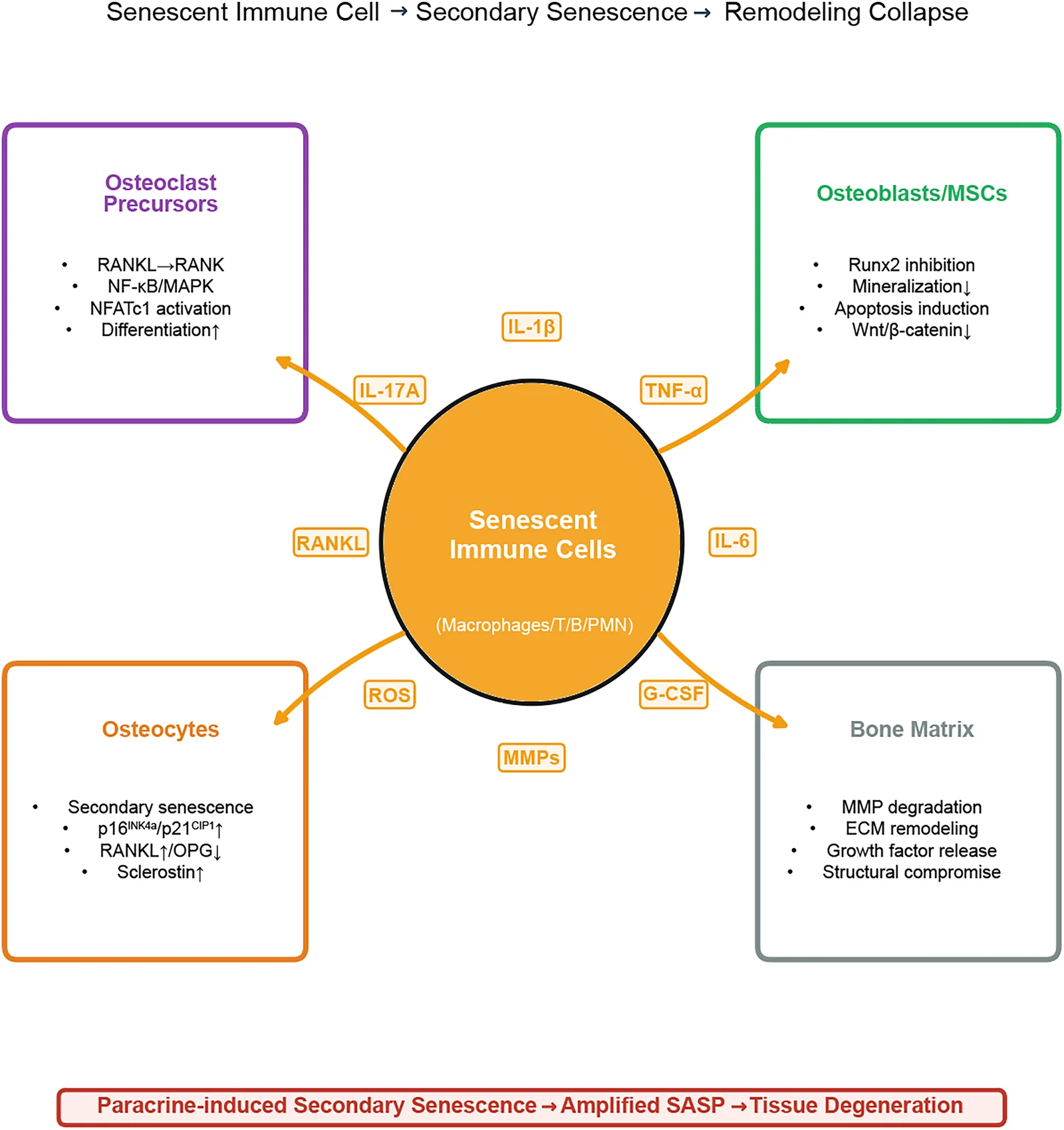
Paracrine SASP propagation in the bone marrow microenvironment. Aged and senescent immune cells including macrophages, T lymphocytes, B lymphocytes, and neutrophils release IL-6, TNF-α, RANKL, ROS, MMPs, and granulocyte colony-stimulating factor (G-CSF). These SASP mediators act directly on osteoclast precursors, osteoblasts, MSCs, osteocytes, and bone matrix to induce secondary senescence, amplify local inflammation, and drive progressive tissue degeneration through a self-perpetuating vicious cycle. ↓ indicates decrease, ↑ indicates increase. Adobe Illustrator 2026 software was used to generate the images.

## 5 Innate immune senescence and bone metabolism

### 5.1 Macrophages

Macrophages are the core cells connecting innate immunity and bone metabolism. A class of tissue-resident macrophages that settle on the endosteal surface and are closely adjacent to osteoblasts, termed osteal macrophages, participate in the coupling regulation of bone remodeling. They support the mineralization function of osteoblasts and promote bone formation; dynamic bone histomorphometric studies have confirmed that their distribution highly overlaps with regions of active bone formation (81). During bone regeneration, osteal macrophages traverse multiple stages including injury response, angiogenesis, and new bone deposition; their absence significantly impairs bone repair capacity (82).

The discovery of osteal macrophages has revised traditional understanding of coupling regulation in bone remodeling. These cells express CD68 and F4/80, cover bone formation surfaces, support matrix mineralization of osteoblasts through secreted signals, and participate in the initiation of the bone formation phase of bone remodeling (81). In bone repair models, the spatiotemporal distribution of macrophages is closely related to the deposition site of new bone; conditional macrophage clearance significantly inhibits both intramembranous and endochondral ossification (82). Bone tissue engineering studies further demonstrate that modulating macrophage phenotype is an effective approach to improve biomaterial osseointegration (83). These findings establish macrophages as positive regulators of bone formation and make the functional consequences of their senescence particularly noteworthy (84).

The functional plasticity of macrophages is reflected in their polarization states. Classically activated M1 macrophages secrete proinflammatory mediators such as TNF-α, IL-1β, IL-6, and nitric oxide, clearing necrotic tissue and initiating inflammation in the early stage of injury. Alternatively activated M2 macrophages secrete IL-10, TGF-β, and vascular endothelial growth factor, participating in inflammation resolution, matrix remodeling, and tissue repair (84). Bone tissue engineering and bone regeneration studies show that the sequential M1-to-M2 transition is a prerequisite for successful fracture healing and osseointegration; an M1 phase that is too short is insufficient to initiate repair, while a prolonged M1 phase impedes osteogenesis (83). At the level of bone homeostasis, the M1 cytokine profile promotes the differentiation of osteoclast precursors into mature osteoclasts, whereas the M2 cytokine profile inhibits osteoclast generation and supports osteogenesis; the balance of macrophage polarization therefore becomes a critical node in bone immune regulation (84).

Macrophage subsets identified as senescent by p21 or p21 and TREM2 expression show decreased phagocytic function. Aging impairs macrophage phagocytosis, chemotactic capacity, and antigen presentation efficiency, while basal inflammatory factor secretion increases, presenting a paradoxical phenotype of proinflammation and functional decline (85). Type 2 cytokine signaling can protect macrophages from senescence; after this signal weakens, macrophages develop p21-mediated senescent phenotypes and promote organismal aging, suggesting that macrophage senescence is actively regulated by the cytokine microenvironment (86). Recent studies have identified a population of senescent macrophages co-expressing p21 and TREM2; these cells expand with age and continuously release inflammatory mediators, serving as an important cellular source driving inflammaging (87). The mechanical microenvironment also participates in regulation: matrix stiffness influences macrophage polarization direction through the Piezo1-YAP axis, and changes in the mechanical properties of aged bone tissue may indirectly shape macrophage phenotypes through this pathway (88). In inflammatory bone disease models, interventions promoting M2 polarization can inhibit osteoclast generation and alleviate bone destruction, highlighting the decisive role of macrophage phenotype in bone metabolic outcomes (89). Overall, macrophages in aging individuals show a pattern of enhanced M1 tendency and impaired M2 function; elevated local levels of IL-1β, IL-6, and TNF-α in bone marrow directly promote osteoclast generation on one hand, and indirectly amplify bone resorption by upregulating RANKL expression in stromal cells and T cells on the other hand. Meanwhile, the reduction of M2-derived IL-10 and TGF-β weakens osteogenic support, and the net effect points toward bone remodeling imbalance (28, 84).

The bone metabolic consequences of macrophage senescence are particularly evident in fracture healing. Fracture healing progresses through the inflammatory phase, soft callus formation phase, hard callus formation phase, and remodeling phase; the intensity and duration of the inflammatory phase determine the quality of subsequent repair (90). In elderly individuals, the inflammatory phase after fracture is prolonged, the M1-to-M2 transition of macrophages is delayed, and the peak expression of inflammatory cytokines is shifted backward, leading to delayed callus mineralization and bone union (91). Single-cell level studies have revealed the presence of osteochondral progenitor cells and neutrophils expressing p21 and multiple senescence markers in fracture hematomas; these senescence-like cells regulate the repair process through SASP factor secretion, and clearance of such cells can accelerate fracture healing in aged mice (92, 93). Immunoengineering strategies targeting the fracture microenvironment, such as biomaterials that deliver immunomodulatory factors in a time-sequential manner, are designed based on understanding of the temporal roles of immune cells (94). These findings directly link immune cell senescence to the clinical challenge of delayed fracture healing in the elderly.

From a mechanistic perspective, the effects of aging on fracture healing span all stages. During hematoma formation, inflammatory cell infiltration patterns change in senescent individuals, with abnormal peak expression of IL-6 and TNF-α (91). During the repair phase, the proliferation and differentiation potential of osteochondral progenitor cells declines, and the accumulation of senescence-like progenitor cells further inhibits endochondral ossification (92). During the remodeling phase, the coupling efficiency between osteoclasts and osteoblasts decreases, and callus remodeling is delayed (90). Clinically, elderly patients with osteoporotic fractures have significantly higher rates of delayed union and nonunion than younger patients, and osseointegration after vertebroplasty and hip fracture surgery is also affected (93). The continuous proinflammatory secretion of senescent immune cells is considered a pathological factor throughout the entire process, while interventions targeting senescent cells in preclinical models can accelerate bone healing, suggesting the intervenability of this link (92, 93).

### 5.2 Neutrophils

Neutrophils are the most numerous innate immune cells in circulation and also the first effector cells to arrive at sites of acute inflammation. Neutrophils exhibit circadian rhythmic aging; freshly released neutrophils and neutrophils aged several hours in circulation differ in phenotype and function, a process regulated by the microbiome through Toll-like receptor and CXCR4 signaling (95, 96). In the context of organismal aging, the chemotactic precision, phagocytic capacity, and intracellular killing efficiency of neutrophils decline, while basal ROS generation and inflammatory mediator release increase. Delayed apoptosis causes them to persist at inflammatory sites, resulting in tissue damage and chronic inflammation (97). This inefficient activation state is considered an important contributor to impaired tissue repair in the elderly.

The relationship between neutrophils and bone metabolism has received increasing attention in recent years. Neutrophils can promote osteoclast differentiation through RANKL expression, ROS release, and formation of neutrophil extracellular traps (NETs). In rheumatoid arthritis, NET-associated protein carbamylation and histone release can induce osteoclast generation and aggravate bone erosion (98). In periodontitis models, neutrophils and osteogenic lineage cells form a pathological neutrophil-osteoblast axis that amplifies local inflammatory bone destruction (99). In contrast to their pro-resorptive effects, neutrophil progenitors in bone marrow have been found to inhibit osteoclast formation, suggesting that the neutrophil lineage has stage-dependent bidirectional regulation of bone metabolism (100). In fracture repair, neutrophils expressing p21 and senescence markers directly participate in repair regulation, and their SASP contributes to the inflammatory microenvironment at the injury site (92). In aging individuals, increased neutrophil ROS generation and decreased phagocytic clearance prolong the inflammatory phase and tilt it toward a pro-resorptive direction, constituting one of the cellular bases for delayed bone repair in the elderly (90, 97).

It should be emphasized that much of the evidence linking neutrophils to bone destruction derives from inflammatory disease contexts, including rheumatoid arthritis and periodontitis, rather than from physiological skeletal aging. These findings establish that neutrophils can mediate bone destruction under inflammatory conditions, but they do not demonstrate that aged neutrophils causally drive age-related bone loss in humans. Direct aging-related evidence for neutrophils remains limited to phenotypic and functional alterations and to the presence of p21-expressing neutrophils in fracture hematomas of aged mice; a causal contribution of neutrophil aging to physiological bone loss therefore awaits dedicated experimental demonstration.

Age-associated neutrophil dysfunction also involves disruption of their circadian regulation. The CXCR2-CXCR4 axis controls neutrophil release from and homing to bone marrow; the microbiome influences the aging rate of neutrophils by modulating this axis (95). During aging, bone marrow neutrophil reserves and release rhythms change, potentially affecting the intensity and duration of inflammatory responses after injury (96). In addition, changes in NET formation tendency of aged neutrophils are associated with bone metabolic diseases; the dual role of NET components as autoantigens and pro-osteoclastic signals warrants further investigation (98). Overall, aged neutrophils participate in age-related bone remodeling imbalance through three pathways: prolonging inflammation, increasing oxidative burden, and providing pro-osteoclastic signals (97, 99).

Clinical evidence also supports the link between neutrophils and bone metabolism. Peripheral neutrophils in patients with chronic inflammatory diseases display functional decline phenotypes similar to aging and are accompanied by decreased bone density (97). In elderly fracture patients, elevated peripheral neutrophil-to-lymphocyte ratio is associated with poor prognosis, reflecting the association between systemic inflammatory burden and bone repair capacity (90). Interventions targeting neutrophils, such as promoting their apoptosis and clearance, inhibiting excessive NET formation, or blocking their RANKL expression, may become adjunct strategies for limiting inflammatory bone destruction (98, 99). The functional coupling between aged neutrophils and other immune cells, such as failed handover with macrophages during the inflammation resolution phase, is an important direction in research on impaired tissue repair in the elderly (96, 97).

### 5.3 NK cells

NK cells clear virus-infected cells and tumor cells through cytotoxic effects and are also important sources of cytokines. NK cells have bidirectional regulatory effects on bone metabolism: their membrane-bound and soluble RANKL expression can promote osteoclast generation, while their secretion of IFN-γ inhibits osteoclast differentiation by inducing degradation of the RANK adaptor protein TRAF6 in osteoclast precursors (101). In multiple myeloma bone disease models, invariant natural killer T cell deficiency leads to impaired regulation of osteoclast generation and aggravated bone destruction, suggesting that the NK cell lineage has an inhibitory surveillance function over osteoclasts (102).

During aging, the number, phenotype, and function of NK cells all undergo characteristic changes. The proportion of CD56^bright^ subsets increases while the cytotoxicity of CD56^dim^ subsets decreases; perforin and granzyme release are reduced, and the expression of NK cell activating receptors is downregulated (103, 104). Functionally weakened NK cells have reduced efficiency in clearing senescent cells and are an important reason for the accumulation of senescent cells *in vivo* (69). For bone metabolism, NK cell aging means weakened IFN-γ-mediated inhibition of osteoclasts, while the diminished ability of NK cells to regulate neutrophil apoptosis delays inflammation resolution (103). The superposition of these two effects tilts the aged bone marrow microenvironment toward a pro-resorptive direction. Given that NK cells are the first line of defense in clearing senescent cells, their functional decline simultaneously exacerbates the accumulation of senescent osteocytes and senescent immune cells in bone tissue (104).

The mechanisms of NK cell aging involve telomere shortening, imbalance between activating and inhibitory receptors, and metabolic adaptation disorders. Lifestyle interventions, including regular exercise and nutritional management, have been shown to partially maintain NK cell function in elderly individuals, suggesting that NK cell immunosenescence has plasticity (104). From a bone metabolism perspective, the interaction between NK cells and other immune cells is also important: NK cells influence inflammation resolution by regulating neutrophil apoptosis and restrain T helper 17 (Th17) responses through IFN-γ secretion; both pathways weaken with aging (102, 103). NK cell functional reconstruction, such as cytokine-induced memory-like NK cells or NK cell adoptive transfer, has progressed in the oncology field and may provide references for bone aging research (69, 104). Direct evidence that NK cell aging causally alters bone metabolism in physiological skeletal aging is likewise limited, and most mechanistic data on NK cells and bone derive from tumor or infection models.

### 5.4 DCs

DCs are professional antigen-presenting cells responsible for connecting innate and adaptive immunity. Within the osteoimmunology framework, DCs share precursor lineage with osteoclasts; under inflammatory conditions, DCs can transdifferentiate into osteoclast-like cells to participate in bone destruction. On the other hand, DCs indirectly influence bone metabolism by presenting antigens to regulate T cell subset differentiation (105). Aged DCs show decreased antigen uptake, processing, and presentation capacity, abnormal costimulatory molecule expression, but increased basal secretion of proinflammatory factors IL-1β, IL-6, and TNF-α, presenting a dysregulated proinflammatory phenotype (44). Under normal conditions, DCs can maintain immune tolerance and indirectly support bone homeostasis by inducing Tregs; the impaired ability of aged DCs to induce Tregs renders this protective loop ineffective (28, 105). DC aging also indirectly exacerbates the aging-related remodeling of the T cell pool by weakening the activation capacity for naive T cells (32). Although direct studies on DC senescence and bone metabolism remain limited, existing evidence is sufficient to establish them as an important node in the network through which immunosenescence affects bone remodeling (29).

The role of DCs in inflammatory bone diseases provides additional clues for their connection with bone metabolism. In rheumatoid arthritis and periodontitis lesions, DC numbers increase and they participate in maintaining local T cell activation, indirectly amplifying osteoclast generation (105). DC-targeted intervention strategies, including modulating DC maturation status and antigen presentation function, have been proposed as new directions for osteoimmune therapy (29, 105). The elevated basal secretion of proinflammatory factors by aged DCs also participates in maintaining the inflammatory aging of the bone marrow microenvironment, superimposing with the proinflammatory changes of macrophages and T cells (44, 85). Future direct studies on DC subsets in aged bone marrow are needed to clarify their weight in bone remodeling imbalance (32). As with neutrophils, evidence implicating DCs in bone destruction derives predominantly from inflammatory disease models, and direct evidence connecting DC aging to bone loss in physiological skeletal aging remains scarce.

## 6 Adaptive immune senescence and bone metabolism

### 6.1 T cell senescence: an overview

T cells are the most extensively studied immune cell population in bone immune regulation. Activated T cells express RANKL and secrete TNF-α, IL-17, and other factors to promote osteoclast generation, while also secreting IFN-γ, IL-4, and other factors to inhibit bone resorption. The net effect of T cells on bone metabolism depends on their subset composition and activation status (23, 106). The abundance of T cells in bone marrow increases with aging, and their phenotype shifts from naive cell dominance to memory and terminally differentiated cell dominance, with corresponding changes in the inflammatory cytokine secretion profile (107). The premature senescence of T cells observed in osteolytic diseases suggests that T cell senescence itself can independently promote bone loss: senescent CD4^+^ and CD8^+^ T cells accelerate secretion of TNF-α, IL-17A, RANKL, and ROS, while downregulating osteogenic signals such as Wnt family member 10b (Wnt10b) (107). Based on such evidence, the concept of immunoporosis has been proposed to describe the association between immune cell senescence and bone fragility, with its proponents arguing that immune system aging acts as an independent driver of age-related bone loss (108).

T cell subset regulation of osteoporosis bone remodeling has multilayered characteristics. Beyond the classical RANKL/RANK/OPG axis, it involves inflammatory cytokine networks, costimulatory molecules, immune checkpoints, and cellular metabolic reprogramming (109). Th17/Treg balance, Th1/Th2 balance, and subsets such as γδT cells and follicular helper T cells show characteristic changes in different types of osteoporosis (106, 109). T cell senescence further pushes these balances toward a pro-resorptive direction: the reduction of naive T cells impairs responses to new antigens, while accumulated terminally differentiated cells continuously secrete pro-bone-resorptive factors (107). The impaired clearance of autoreactive T cells due to thymic involution makes the local bone marrow more prone to low-grade inflammation against self-antigens (49). These changes collectively constitute the pro-resorptive phenotype of the aged bone marrow T cell compartment.

### 6.2 Th17 cells

Th17 cells are a T cell subset that clearly promotes osteoclast generation. In 2006, Sato et al. demonstrated that Th17 cells induce RANKL expression in osteoblasts and synovial fibroblasts through IL-17A secretion and strongly promote osteoclast differentiation through an IL-17A-mediated inflammatory amplification loop, thereby directly linking T cell activation to bone destruction (110). The pathogenicity of Th17 cells has been fully validated in inflammatory bone diseases such as rheumatoid arthritis; their secreted IL-17A, IL-21, and IL-22 synergistically enhance local inflammation and bone erosion (111). IL-17A can also amplify the response of osteoclast precursors to RANKL by activating NF-κB and MAPK signaling and inhibit the differentiation and mineralization functions of osteoblasts (112). Calcium/calmodulin-dependent protein kinase IV and other signaling nodes simultaneously regulate Th17 cell activation and osteoclast generation; inhibition of this pathway produces dual anti-bone-destruction effects (113).

Aging is accompanied by a systemic shift in Th17/Treg balance. In the aged bone marrow microenvironment, elevated levels of IL-1β, IL-6, and IL-23 combined with relative IL-2 deficiency create a cytokine milieu that favors polarization of naive CD4^+^ T cells toward Th17 rather than maintenance of Tregs (114, 115). In estrogen deficiency models, Th17 cell expansion has been proven to be an important mechanism of bone loss; inhibiting Th17 differentiation or blocking IL-17A can attenuate ovariectomy-induced bone mass decline (114). Cross-sectional studies in postmenopausal osteoporosis patients similarly show elevated peripheral Th17 proportions and increased IL-17A levels, negatively correlating with bone density (116, 117). Therefore, age-related Th17 expansion constitutes a key adaptive immune mechanism linking immunosenescence to enhanced bone resorption.

Th17 cell differentiation depends on the combined signals of IL-6, IL-23, TGF-β, and IL-1β; the transcription factor RORγt is their lineage-determining factor (110). The elevated levels of IL-1β and IL-6 in aged bone marrow precisely provide the cytokine environment for Th17 polarization (115). There is also direct metabolic and signaling interaction between Th17 cells and osteoclasts: Th17 cells highly express RANKL and can express auxiliary factors for osteoclast generation; their secreted IL-17A induces osteogenic lineage cells to produce RANKL and proinflammatory chemokines, recruiting more osteoclast precursors (112). The bone immune network framework of RANKL convergence integrates macrophage activation, T cell imbalance, and cytokine crosstalk into a unified pro-resorptive network, with Th17 as its core node (117). Upstream strategies targeting Th17 differentiation and blocking IL-17A itself have been validated as effective in inflammatory bone diseases, and their value in senile osteoporosis warrants exploration (113, 118).

### 6.3 Tregs

Tregs inhibit osteoclast generation and maintain bone homeostasis through multiple mechanisms. Zaiss et al. first demonstrated that Tregs can inhibit osteoclast differentiation in a cell contact-dependent manner; their effect partially depends on cytotoxic T-lymphocyte-associated protein 4 blocking costimulatory signals on osteoclast precursors (119). IL-10 and TGF-β secreted by Tregs directly inhibit the differentiation program of osteoclast precursors, while indoleamine 2,3-dioxygenase-mediated tryptophan metabolism also plays a role (120, 121). In inflammatory bone diseases, insufficient Treg number or function correlates with the degree of bone destruction, and restoring Treg function can alleviate bone erosion (122).

The effect of aging on Tregs has two sides: the number of peripheral Tregs in elderly individuals often does not decrease and may even increase, but their suppressive function and tissue repair capacity decline (123). Senescent Tregs display the phenotypic characteristic of Foxp3^+^CD28^−^; abnormal expression of cholesterol metabolism-related molecules such as SOAT2 suggests impaired metabolic adaptation (124). More pathologically significant is Treg lineage instability: in inflammatory environments, some Tregs lose Foxp3 expression and convert into exFoxp3 Th17 cells, which in turn secrete IL-17A, TNF-α, and RANKL, transforming from bone-protective cells into bone-destructive effector cells. This conversion has been confirmed in rheumatoid arthritis joint destruction (122, 125, 126). The functional decline of Tregs weakens their inhibition of osteoclasts and their restraint of Th17 expansion, causing the local immune negative regulatory network in bone marrow to collapse (121). Treg-based therapeutic strategies have shown potential in the field of bone regeneration; local administration of Tregs can promote tissue healing, and induced Treg-derived extracellular vesicles can inhibit CD4^+^ T cell-mediated inflammation and alleviate bone loss through a CD73-adenosine-dependent mechanism (127, 128).

The bone-protective mechanisms of Tregs have multilayered characteristics. Beyond cytotoxic T-lymphocyte-associated protein 4-mediated contact-dependent inhibition, IL-10 secreted by Tregs inhibits NFATc1 induction in osteoclast precursors, while TGF-β interferes with RANKL downstream signaling (119, 120). Tregs also indirectly limit effector T cell activation by inhibiting antigen-presenting cell function, reducing the production of pro-bone-resorptive factors at the source (121). The cellular basis for the functional decline of senescent Tregs includes mitochondrial dysfunction, impaired metabolic adaptation, and decreased tissue residency capacity; their diminished tissue repair function has been demonstrated in systems such as the nervous system (123, 124). In bone tissue, the consequences of Treg senescence are dual: loss of both direct inhibition of osteoclasts and restraint of Th17 expansion and macrophage proinflammatory polarization (115, 122).

### 6.4 CD8^+^ and memory T cells

CD8^+^ T cell regulation of bone metabolism is also bidirectional; their effect depends on the cytokine secretion profile and the microenvironment in which they reside. Under physiological conditions, CD8^+^ T cells can support bone formation by secreting osteogenic factors such as Wnt10b; under pathological conditions, activated CD8^+^ T cells secrete TNF-α and RANKL to promote bone resorption (129). Aging is accompanied by reduced naive CD8^+^ T cells and accumulation of terminally differentiated CD28^−^ CD8^+^ T cells; the latter have diminished cytotoxicity but enhanced inflammatory factor secretion, accompanied by increased mitochondrial ROS generation (130, 131). Highly differentiated T cell subsets accumulate in tissues with age and promote multi-organ tissue degeneration through persistent proinflammatory secretion; bone tissue is no exception (131).

The position of memory T cells in bone immune regulation has gradually become clear in recent years. Bone marrow is an important reservoir for memory T cells; during aging, the phenotype and cytokine secretion profile of bone marrow memory T cells change (51). The tumor necrosis factor superfamily member LIGHT is expressed by activated T cells and can participate in estrogen deficiency-induced bone loss by influencing osteoclast generation and bone remodeling; LIGHT-deficient mice show obvious abnormalities in bone remodeling (132, 133). Under estrogen deficiency conditions, memory T cells are activated and impair bone integrity through different mechanisms in cortical and trabecular bone compartments, suggesting that memory T cells are important effector cells connecting sex hormone changes to bone loss (134). T cell-derived DKK1 is an inhibitor of Wnt signaling; mice with T cell-specific DKK1 deficiency show increased bone mass and resistance to estrogen deficiency-induced bone loss, proving that T cells can directly inhibit bone formation by secreting Wnt inhibitors in the context of aging and hormonal changes (135).

The functional consequences of CD8^+^ T cell senescence are context-dependent. In multiple myeloma models, mitochondrial ROS-induced CD8^+^ T cell senescence weakens antitumor immunity; inhibition of IRE1α can simultaneously reverse T cell senescence and tumor progression, suggesting that metabolic intervention can reshape the function of senescent CD8^+^ T cells (130). In the osteoporosis context, imbalance of CD8^+^ T cell subsets correlates with decreased bone density, with mechanisms involving altered RANKL expression, IFN-γ secretion changes, and regulation of osteoclast generation (129). Extremely differentiated T cell subsets accumulate in tissues such as bone marrow with age, and their persistent proinflammatory secretion is considered a driver of tissue degeneration (52, 131). Interventions targeting these cells may become a new approach for improving bone metabolism in the elderly.

### 6.5 B cells

B cells occupy a unique position in the bone immune axis, with functions extending far beyond antibody production. B cells are the primary source of OPG in bone marrow; under physiological conditions, they inhibit osteoclast generation through OPG secretion and exert bone-protective effects. However, activated and senescent B cells can express RANKL and TNF-α, switching to promote bone resorption (136, 137). The effects of aging on B cells manifest as reduced naive B cell output, decreased antibody diversity, and accumulation of age-associated B cells (ABCs) (138, 139). The transcription factor ZEB2 has been shown to drive ABC formation; these cells have proinflammatory and autoreactive characteristics and are associated with multiple aging-related diseases (140).

The bone metabolic effects of senescent B cells undergo a transformation from protection to destruction. The classic study by Li et al. showed that, compared with young mice, bone marrow B cells in aged mice increased OPG secretion by approximately 25-fold, presenting a compensatory protective response to bone loss. However, while young mouse B cells showed almost no detectable RANKL production, aged mouse B cells produced significant levels of RANKL, and the net effect of RANKL expression ultimately becomes dominant (22). With increasing age, total B cell numbers decline, and their bone-protective capacity is insufficient to offset the pro-resorptive effects of RANKL and inflammatory factors (136). In severely CD4^+^ T cell-depleted HIV-infected individuals, expansion of immature and transitional B cells is significantly associated with bone loss; these B cells highly express RANKL, suggesting that B cell subset imbalance itself is an independent risk factor for bone loss (55). In addition, senescent B cells can secrete G-CSF to promote osteoclast precursor proliferation and inhibit osteoblast differentiation through NF-κB and extracellular signal-regulated kinase signaling (28, 137). B cells and T cells influence each other during aging; changes in B cells can accelerate T cell immunosenescence, forming a degenerative network of the adaptive immune system as a whole (141).

From an evolutionary and physiological perspective, the function of B cells in secreting OPG has clear bone-protective significance, which is more prominent under stress conditions. Bone mass changes in B cell-deficient animals and bone mass recovery after B cell reconstitution demonstrate that B cells are active regulators of bone homeostasis rather than bystanders (136). The accumulation of ABCs during aging deserves particular attention: these cells highly express T-bet, secrete proinflammatory factors, and respond poorly to conventional B cell activation signals; their expansion is associated with chronic inflammation and autoantibody production (139, 140). Although direct studies on ABCs and bone metabolism remain limited, their proinflammatory characteristics suggest potential participation in the inflammatory remodeling of the aged bone marrow microenvironment (141). Overall, the net effect of B cell senescence on bone metabolism depends on the algebraic sum of decreased OPG secretion, increased RANKL secretion, and proinflammatory factor release; existing evidence points toward a pro-resorptive direction (22, 137).

## 7 Immunosenescence-derived mediators of bone remodeling

### 7.1 Proinflammatory cytokines

The effects of the major SASP factors on osteoblasts and osteoclasts are summarized in **Table 1**. Senescent immune cells and senescent bone cells release SASP factors that constitute a pro-resorptive, anti-synthetic bone marrow microenvironment. The effects of the main effector molecules on osteoblasts and osteoclasts have been relatively well elucidated (142). IL-6 and its family members indirectly promote osteoclast generation by enhancing RANKL expression in osteoblasts and stromal cells, while simultaneously inhibiting osteoblast matrix synthesis and mineralization; under the background of inflammaging, sustained IL-6 elevation is an independent risk factor for age-related bone loss (142, 143). The effects of TNF-α are more extensive: it can directly enhance the responsiveness of osteoclast precursors to RANKL, promote osteoclast generation, and prolong the survival of mature osteoclasts; at the same time, TNF-α induces osteoblast apoptosis, inhibits Runx2 activity, and reduces collagen synthesis, disrupting bone remodeling balance from both the resorption and formation directions (144, 145). IL-1β is a potent promoter of bone resorption; it has an autocrine amplification loop with TNF-α, can synergistically upregulate RANKL, and activates the NLRP3 inflammasome, forming positive feedback between inflammation and bone resorption (146, 147). Inhibition of IL-1β or the NLRP3 inflammasome in experimental models can significantly reduce osteoclast generation and bone resorption (148). The role of IL-17A has been detailed previously; it promotes osteoclast differentiation through upregulating RANKL and activating the NF-κB/MAPK/NFATc1 pathway, and is the core effector molecule of Th17-mediated bone destruction (110, 112).

**Table 1:** Effects of major SASP factors on osteoblasts and osteoclasts.

SASP factor	Main cellular sources	Effects on osteoclasts	Effects on osteoblasts
IL-6	Senescent macrophages, T cells, osteocytes	Upregulates RANKL expression, promotes osteoclast differentiation	Inhibits matrix synthesis and mineralization
TNF-α	Senescent T cells, macrophages	Enhances RANKL sensitivity, prolongs osteoclast survival	Induces apoptosis, inhibits Runx2 activity
IL-1β	Senescent macrophages, DCs	Activates NLRP3 inflammasome, amplifies osteoclast generation	Interferes with Wnt/β-catenin signaling, inhibits differentiation
IL-17A	Th17 cells, exFoxp3 Th17 cells	Promotes differentiation via RANKL and NF-κB/MAPK/NFATc1 pathway	Inhibits osteoblast differentiation and mineralization
RANKL	Senescent B cells, T cells, osteocytes	Directly binds RANK, drives osteoclast terminal differentiation	No direct effect
ROS	Aged neutrophils, CD8^+^ T cells	Activates NF-κB to promote osteoclast generation	Induces apoptosis, inhibits matrix protein synthesis
MMP	Senescent stromal cells, immune cells	No direct effect	Degrades extracellular matrix, compromises bone structural integrity
G-CSF	Senescent B cells	Promotes osteoclast precursor proliferation	No direct effect

The IL-6 cytokine family has member-specific effects on bone. Both classical and trans-signaling of IL-6 can enhance RANKL expression, while family members such as oncostatin M and leukemia inhibitory factor also participate in regulation on the bone formation side; the balance among family members determines the net effect (143). The synergy between TNF-α and RANKL is particularly critical: TNF-α induces osteoclast precursors to express RANK and enhances their sensitivity to RANKL, and low concentrations of TNF-α can significantly amplify RANKL-induced osteoclast generation (144). The autocrine loop between IL-1β and TNF-α maintains inflammatory signals self-sustaining; mononuclear macrophages are both the source and target of these factors (146). The receptor signals of these cytokines converge on NF-κB and NFATc1 at the osteoclast precursor level, explaining the convergence of pro-bone-resorptive effects of multiple inflammatory factors (17, 147). The continuous release of the above factors by senescent immune cells exerts persistent influence on bone remodeling through this convergence mechanism (142).

### 7.2 ROS and oxidative stress

ROS are a common molecular hub of immune senescence and bone aging. Mitochondrial dysfunction in senescent immune cells leads to increased ROS generation. On one hand, ROS act as signaling molecules to activate NF-κB and promote osteoclast differentiation; on the other hand, they induce osteoblast and osteocyte apoptosis and inhibit bone matrix protein synthesis (149). Oxidative stress also alters the antioxidant defense and survival status of bone cells by promoting nuclear translocation of FoxO transcription factors, and is considered an important supplement to the estrogen-centric hypothesis in osteoporosis pathogenesis (10). In bone tissue, oxidative stress and inflammation mutually amplify each other: inflammatory factors stimulate ROS generation, and ROS in turn activate inflammasomes and the NF-κB pathway, forming a vicious cycle that maintains chronic inflammation (150). Research in a diabetic periodontitis model showed that senescent CD4^+^ T cells drive inflammatory bone destruction through the JAK-STAT-ROS-p38 MAPK axis, directly linking T cell senescence, oxidative stress, and bone loss (151). Antioxidant interventions, such as promoting mitophagy and mitochondrial biogenesis to alleviate MSC senescence, can restore osteogenic capacity in animal models, suggesting that the ROS pathway is intervenable (152).

MMPs are an easily overlooked effector component of SASP. MMP-3, MMP-9, and others are released by senescent cells, degrade non-collagen components of bone matrix, compromise bone structural integrity, and alter the local microenvironment by releasing growth factors stored in the matrix (66, 76). Extracellular vesicles as a recently recognized SASP component have received attention: senescent cell-derived extracellular vesicles carry miRNA and protein cargo and can transmit senescence signals remotely (67). In the bone marrow microenvironment, such vesicle-mediated communication may explain the spatial propagation of senescent phenotypes within bone tissue (47).

### 7.3 Paracrine SASP propagation

The age-associated phenotypes, proposed skeletal effects, molecular mediators, and evidence levels of the major immune cell populations are summarized in **Table 2**. The harm of SASP lies not only in the direct effects of its constituent factors but also in its ability to induce secondary senescence. IL-6, IL-1β, TNF-α, TGF-β family molecules, and extracellular vesicles secreted by senescent immune cells can act on neighboring MSCs, osteoblast precursors, and osteocytes, inducing them to enter a senescent state and causing the senescent phenotype to propagate along cellular networks (67, 76). The limited space of bone marrow determines the close contact between immune cells, hematopoietic cells, and skeletal cells, so paracrine-propagated senescence signals are significantly amplified in the bone marrow microenvironment (47). The observation that senescence markers decrease overall in bone tissue after senescent cell clearance, together with the experimental result that local clearance can only partially replicate the benefits of systemic clearance, supports the remote influence of systemic senescence burden on bone tissue (72, 80). The resulting picture is that immune senescence and bone cell senescence are mutually causal, jointly maintaining a proinflammatory, pro-resorptive, and anti-synthetic bone marrow microenvironment that drives the chronic progression of bone remodeling imbalance.

**Table 2:** Immune cell populations in age-related bone remodeling: phenotypes and levels of evidence.

Immune cell population	Major age-associated or senescence phenotype	Proposed effect on bone cells	Key molecular mediators	Disease or model context	Level of evidence
Macrophages	Enhanced M1 proinflammatory polarization, impaired phagocytosis and efferocytosis; p21-positive or p21 and TREM2 co-expressing senescent subsets	Promote osteoclast generation; weaken osteogenic support and fracture repair	TNF-α, IL-1β, IL-6, IL-10, TGF-β	Aged mice, ovariectomy models, fracture healing, inflammatory bone disease	Human observational, animal, *in vitro*
Neutrophils	Impaired chemotaxis and phagocytosis, increased ROS, delayed apoptosis; disrupted circadian aging	Promote osteoclast generation via RANKL, ROS, and NETs; prolong the inflammatory phase	RANKL, ROS, NET-associated carbamylated proteins and histones	Rheumatoid arthritis, periodontitis, fracture hematoma; direct physiological aging evidence limited	Human observational, animal, *in vitro*
NK cells	Reduced cytotoxicity, decreased perforin and granzyme release, reduced IFN-γ production	Weakened inhibition of osteoclasts; impaired clearance of senescent cells	IFN-γ, RANKL	Aged individuals, multiple myeloma models	Human observational, animal
Dendritic cells	Impaired antigen uptake and presentation; increased basal IL-1β, IL-6, and TNF-α secretion	Indirect promotion of osteoclast generation via T cell activation; weakened Treg induction	IL-1β, IL-6, TNF-α	Rheumatoid arthritis, periodontitis; direct aging evidence scarce	Human observational, animal, *in vitro*
Th17 cells	Expansion with age, increased Th17/Treg ratio	Promote osteoclast generation; inhibit osteoblast differentiation and mineralization	IL-17A, IL-21, IL-22, RANKL	Estrogen deficiency models, postmenopausal osteoporosis patients	Human observational, animal, *in vitro*
Tregs	Functional decline, Foxp3-positive CD28-negative phenotype, lineage instability with exFoxp3 conversion	Weakened inhibition of osteoclasts; loss of restraint on Th17 expansion	IL-10, TGF-β, cytotoxic T-lymphocyte-associated protein 4, CD73-adenosine	Aged mice, rheumatoid arthritis, periodontitis	Human observational, animal
CD8+ and memory T cells	Accumulation of terminally differentiated CD28-negative cells, increased mitochondrial ROS	Promote bone resorption via TNF-α and RANKL; context-dependent support of formation via Wnt10b	TNF-α, RANKL, Wnt10b, DKK1, LIGHT	Estrogen deficiency models, cytomegalovirus infection, multiple myeloma	Human observational, animal
B cells	Reduced naive output, accumulation of age-associated B cells, increased RANKL/OPG ratio	Net shift from bone protection to bone resorption	OPG, RANKL, G-CSF, TNF-α	Aged mice, HIV-associated bone loss, postmenopausal osteoporosis	Human observational, animal, *in vitro*

From an intervention perspective, interrupting the paracrine propagation of SASP has multiple strategic options: blocking signals of key propagation mediators such as IL-6 and IL-1β; inhibiting transcriptional regulatory nodes of SASP such as mTOR; and restoring immune surveillance for senescent cell clearance (67, 69). Among these, restoring immune surveillance is particularly fundamental because it targets the upstream cause of senescent cell accumulation (69, 70). However, senescent immune cells themselves are the main executors of this surveillance function, constituting the central paradox that bone aging intervention must confront, and explaining why combined strategies of immune reconstitution and senescent cell clearance are receiving attention (32, 153).

Direct causal evidence in physiological skeletal aging is strongest for macrophages and T cell subsets and remains limited for neutrophils, dendritic cells, and NK cells; rows drawing on inflammatory disease models are indicated in the corresponding column. Marker-defined cellular senescence has been demonstrated for macrophage subsets, T cell subsets, and B cells in aged bone marrow, whereas for neutrophils, dendritic cells, and NK cells the phenotypes listed are age-associated functional alterations rather than bona fide cellular senescence.

Viewed as a whole, the evidence summarized above supports a network model rather than a cell-by-cell model of immune-skeletal aging. Senescent myeloid cells and senescent T and B cells converge on osteoclast precursors through RANKL, TNF-α, IL-6, IL-1β, IL-17A, and ROS, while simultaneously suppressing osteoblast lineage commitment of skeletal stem and progenitor cells and the mineralization activity of mature osteoblasts. Osteocytes, as the principal physiological source of RANKL, integrate these immune-derived signals with mechanical and endocrine inputs and, once senescent, contribute their own SASP to the local milieu. This arrangement creates reciprocal reinforcement: senescent immune cells drive osteoclastogenesis and impair osteogenesis, senescent osteocytes and stromal cells sustain the proinflammatory niche that maintains immune aging, and declining immune surveillance further permits the accumulation of senescent cells of both lineages. The shared bone marrow microenvironment, in which hematopoietic and skeletal cells are separated only by thin bone surfaces and a dense sinusoidal network, provides the structural basis that couples these loops and converts local immune aging into a skeletal phenotype. The therapeutic implication of this network view, namely that simultaneous targeting of multiple nodes may be required for durable benefit, is discussed in Section 10.

## 8 Hormonal regulation of immune-bone crosstalk

Beyond local cellular interactions, systemic factors including hormonal changes, microbial dysbiosis, and chronic infections converge on the immune-bone axis to modulate skeletal homeostasis (**Figure 3**).

**Figure 3:**
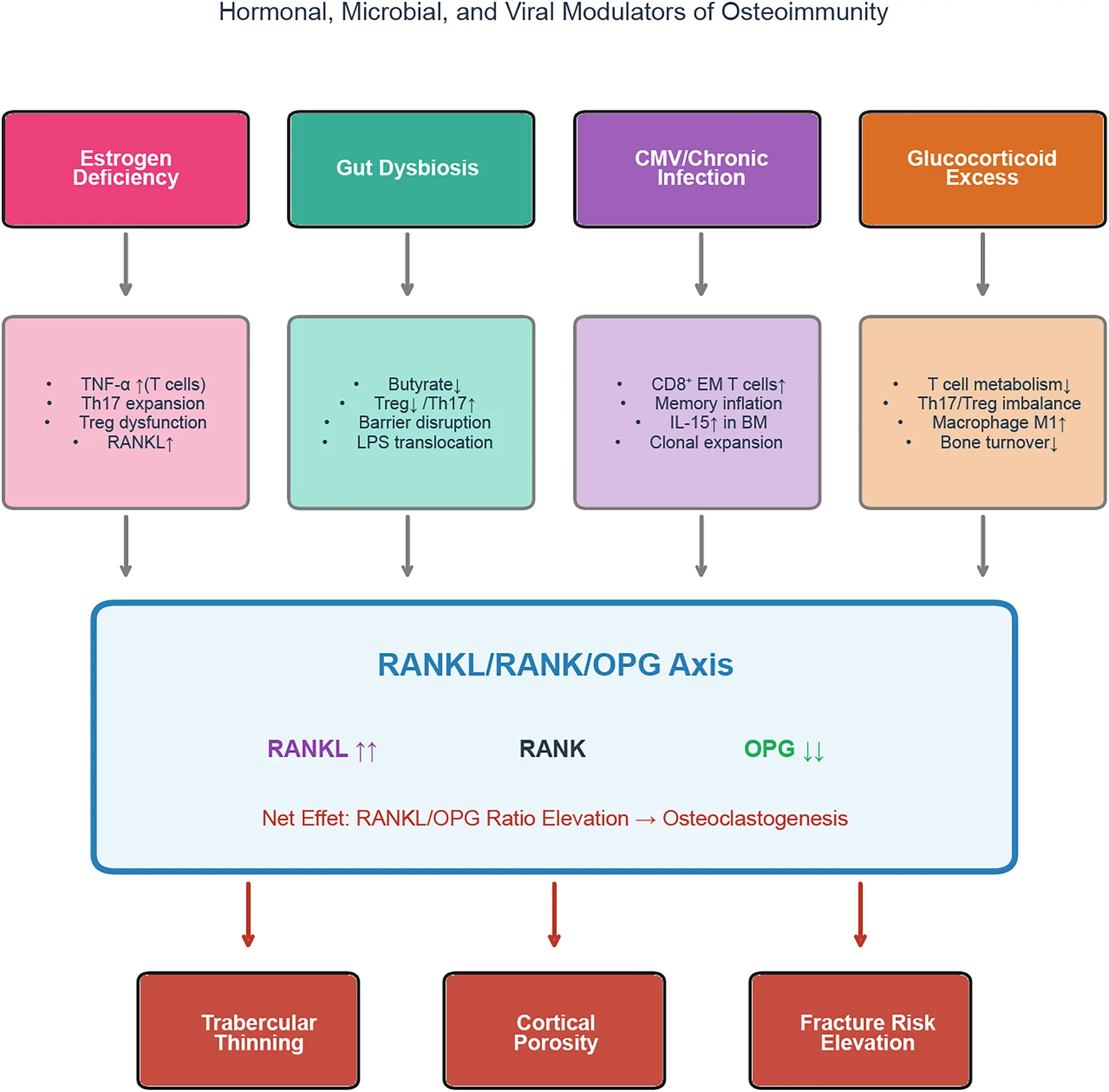
Systemic regulation of the immune-bone axis. Estrogen deficiency, gut dysbiosis, chronic viral infection, and glucocorticoid excess converge on bone marrow immunity by altering T cell subset composition, macrophage polarization states, and inflammatory mediator profiles. These systemic triggers ultimately dysregulate the RANKL/RANK/OPG axis, elevating the RANKL/OPG ratio and driving sustained osteoclast-mediated bone resorption and progressive structural deterioration. ↓ indicates decrease, ↑ indicates increase. Adobe Illustrator 2026 software was used to generate the images.

### 8.1 Estrogen deficiency

Estrogen deficiency is the classic cause of postmenopausal osteoporosis, and its bone loss effects are largely mediated through the immune system. The pioneering study by Cenci et al. demonstrated that estrogen deficiency induces bone loss by enhancing T cell production of TNF-α, and nude mice lacking T cells are resistant to ovariectomy-induced bone loss (154). Subsequent studies elucidated that estrogen deficiency increases the number of TNF-producing T cells in bone marrow, with mechanisms involving enhanced antigen presentation, upregulated costimulatory signals, and IL-7-dependent T cell expansion (155, 156). TNF-α directly enhances the response of osteoclast precursors to RANKL on one hand and inhibits osteoblast function on the other, constituting the core immune mechanism of estrogen deficiency-induced bone loss (154). Manolagas proposed that the pathogenesis of osteoporosis should be expanded from a single estrogen-centric perspective to a comprehensive model involving both aging and oxidative stress, in which the age-related changes of the immune system and bone marrow microenvironment occupy important positions (10).

Recent studies have further refined the cellular details of the estrogen-immune-bone axis. Estrogen deficiency can activate memory T cells, which impair bone integrity through different mechanisms in cortical and trabecular bone compartments (134); Th17 cell expansion and Treg functional impairment jointly participate in bone loss after estrogen withdrawal (114); T cell-derived DKK1 inhibits Wnt signaling, exacerbating imbalance on the bone formation side (135); the LIGHT/tumor necrosis factor superfamily member LIGHT pathway provides another molecular channel for T cell regulation of estrogen deficiency-induced bone loss (132). From the perspective of immunosenescence, the abrupt decline of estrogen after menopause superimposes on age-related immunosenescence: estrogen itself has effects in inhibiting M1 polarization, maintaining Treg function, and limiting Th17 expansion, so its deficiency equals the removal of constraints on the proinflammatory immune pattern, causing aging-related immune imbalance to accelerate sharply (116, 157).

### 8.2 Postmenopausal osteoporosis

Immunophenotype studies in postmenopausal osteoporosis patients provide human evidence for the above mechanisms. Patients show expansion of proinflammatory monocyte subsets in peripheral blood and bone marrow; monocytes expressing thrombospondin 1 have been shown to be drivers of osteoclast generation (158); the Th17/Treg ratio is elevated, and serum IL-17A, TNF-α, and IL-6 levels negatively correlate with bone density (115, 159); peripheral immune cell migration and intestinal flora composition are also altered, suggesting the existence of systemic immune disorders (160). As early as 2005, scholars discussed the connection between osteoporosis, inflammation, and aging, proposing that senile osteoporosis has characteristics of an immune-mediated disease (161). The current view holds that postmenopausal osteoporosis is the product of the synergistic action of estrogen deficiency, immunosenescence, and inflammaging; simply supplementing calcium and vitamin D or simply inhibiting bone resorption cannot fully correct its immunological basis (116). This understanding has driven exploration of interventions targeting immune links, including modulating the Th17/Treg balance and immunotherapy strategies targeting the Breg-Treg axis (118, 162).

Postmenopausal osteoporosis and senile osteoporosis have both overlapping and distinct immunological bases. The former is dominated by acute immune activation triggered by estrogen withdrawal, with T cell TNF-α production and Th17 expansion as core events (114, 154); the latter is dominated by the chronic accumulation of immunosenescence and inflammaging, with the roles of SASP and senescent cell burden being more prominent (72, 161). Clinical observations support this distinction: rapid bone loss in the early postmenopausal period responds well to anti-resorptive therapy, while bone loss in very elderly patients is accompanied by more significant bone marrow fat infiltration and immune composition changes (7, 8). Distinguishing the immunological mechanisms of the two types of osteoporosis helps develop age- and etiology-specific treatment strategies (116, 158).

### 8.3 Parathyroid hormone

Parathyroid hormone (PTH) has dosing pattern-dependent bidirectional effects on bone metabolism: intermittent administration promotes bone formation, while sustained elevation promotes bone resorption (163, 164). The bone effects of PTH also require immune cell participation. T cells are necessary mediators of the bone-forming effects of intermittent PTH administration; Wnt10b produced by T cells promotes osteoblast proliferation and differentiation under PTH stimulation; while under sustained PTH elevation, T cell-secreted TNF-α and RANKL participate in bone resorption (165, 166). Changes in T cell subsets and cytokine profiles in parathyroid disease states are associated with abnormal bone metabolism, suggesting the importance of the PTH-immune-bone axis under pathological conditions (166). Aging-related changes in T cell composition may therefore affect the body’s responsiveness to PTH analogs, a variable worthy of attention in the treatment of senile osteoporosis (28).

The interaction between PTH and the immune system is also reflected in its regulation of Treg and Th17 balance. Intermittent PTH administration can affect the composition and functional status of T cells in bone marrow, while sustained PTH elevation promotes T cell secretion of TNF-α, exacerbating bone resorption (164, 165). These findings suggest that when PTH analogs are used in elderly patients, the senescence status of their immune system may be a source of efficacy differences (163). Optimizing dosing regimens from an osteoimmunology perspective, such as selecting bone-forming drugs based on immune status assessment, is a potential direction for precision anti-osteoporosis therapy (166).

### 8.4 Glucocorticoids

Glucocorticoid-induced osteoporosis is the most common secondary osteoporosis. Excess glucocorticoids directly inhibit osteoblast proliferation and differentiation, induce their apoptosis, prolong osteoclast survival, and inhibit the mechanosensory function of osteocytes (167, 168). At the immune level, glucocorticoids inhibit T cell proliferation and IL-2 production, alter the Th17/Treg balance, and reshape the immune-metabolic status of the bone marrow microenvironment. Studies show that excess glucocorticoids suppress mouse bone turnover by regulating the immune metabolism of the skeletal microenvironment, with metabolic reprogramming of macrophages and T cells participating (169). Elderly populations are both common users of glucocorticoids and in a background of immunosenescence; the superposition of the two significantly increases bone loss risk, and clinical management needs to simultaneously consider bone protection and immune control of the underlying disease (168).

The extensive immunosuppressive effects of glucocorticoids make their bone metabolic effects more complex. On one hand, glucocorticoids inhibit T cell proliferation and proinflammatory factor production, which theoretically could alleviate inflammatory bone resorption; on the other hand, their direct inhibition of osteoblasts and prolongation of osteoclast survival dominate, with a net effect of rapid and significant bone loss (167). Skeletal microenvironment immune metabolism studies show that glucocorticoids reshape the metabolic landscape of bone marrow immune cells and skeletal cells, suppressing overall bone turnover (169). In elderly patients on long-term glucocorticoid therapy, fracture risk is compounded by immunosenescence factors; preventive strategies combining calcium, vitamin D, and anti-resorptive agents have become standard management (168).

## 9 Gut microbiota-immune-bone axis

The gut microbiota indirectly influences bone metabolism by regulating the immune system, constituting the emerging research direction of the gut-immune-bone axis (170). Microbiota and their metabolites can shape T cell subset differentiation, regulate systemic inflammation levels, and affect nutrient absorption. The landmark study by Tyagi et al. demonstrated that the microbial metabolite butyrate upregulates Wnt10b expression in bone-forming cells through a Treg-mediated mechanism, stimulating bone formation; this effect depends on Treg regulation of CD8^+^ T cells, revealing the complete pathway from microbiota metabolites to immune cells to bone formation (171). Aging is accompanied by decreased gut microbiota diversity and compromised barrier function; changes in microbiota composition affect bone mass through immune cell mediation: in postmenopausal osteoporosis patients, gut microbiota disturbance coexists with abnormal peripheral immune cell migration (160); immune cells are considered key mediators of information transfer between microbiota and bone (172, 173).

In the framework of aging-related bone diseases, the significance of the microbiota-bone axis lies in its interaction with immunosenescence (174). Microbiota products regulate macrophage polarization, Th17/Treg balance, and systemic inflammaging levels; conversely, immunosenescence alters intestinal immune surveillance and affects microbiota homeostasis (175). Microbiota-targeted interventions, such as probiotics, synbiotics, and dietary fiber, have shown potential in improving bone density and reducing inflammation in animal models and some clinical pilot studies (176). Microbiota regulation may also indirectly affect osteoporosis occurrence and treatment response through pathways such as the brain-bone axis (177). Evidence in this field is still accumulating, but its value in integrating immunosenescence, systemic inflammation, and bone metabolism into a unified network is already apparent.

Estrogen, gut microbiota, and bone immunity interact with each other: estrogen levels affect microbiota composition and intestinal barrier function, microbiota metabolites regulate the Th17/Treg balance and systemic inflammation, ultimately acting on bone remodeling (157, 178). The disturbance of this network after menopause suggests that microbiota-targeted interventions may complement hormone replacement or anti-resorptive therapy (176).

Evidence for microbiota intervention is moving from animal experiments toward clinical application. Pilot randomized controlled trials of polyphenol-rich foods such as blackcurrant improving postmenopausal bone health through microbiota modulation, and reports of synbiotic medical foods improving intestinal barrier and immune status, provide preliminary human evidence for the intervenability of this axis (176). The deterioration of the microbiota-immune-bone axis during aging is synchronized with musculoskeletal system degeneration, suggesting that combined intervention of microbiota and immunity may simultaneously improve bone and muscle function (174, 175). Of course, the bone-protective effects of microbiota intervention still require validation in large-sample randomized controlled trials (170).

## 10 Therapeutic strategies targeting immunosenescence

Based on the close relationship between immunosenescence and bone metabolism, interventions relevant to age-related bone loss can be organized along three conceptual categories according to their mechanism of action: conventional bone-targeted drugs, immune-targeted interventions, and strategies directed at the bone marrow microenvironment as a whole (109, 119). These categories are complementary rather than sequential; they address different dominant mechanisms and may be combined according to disease stage, inflammatory burden, and senescent cell load. The early rapid bone loss stage is generally managed with anti-resorptive therapy; patients with prominent inflammatory features may be considered for concomitant cytokine blockade; and very elderly patients with a high senescent cell burden are potential candidates for investigational senolytic approaches (179, 180). The three categories differ markedly in their level of evidence and clinical readiness. The first category comprises therapies approved for osteoporosis with well-established fracture reduction. The second category includes approved biologic agents whose bone-protective effects are supported mainly in inflammatory disease populations, together with cell-based strategies that remain experimental; their benefit in elderly osteoporosis patients remains under study. The third category consists largely of preclinical or early-phase strategies that target immunosenescence directly and remain investigational. We summarize this stratification in **Figure 4** and **Table 3**, and we indicate the clinical status of each strategy in the corresponding sections.

**Figure 4:**
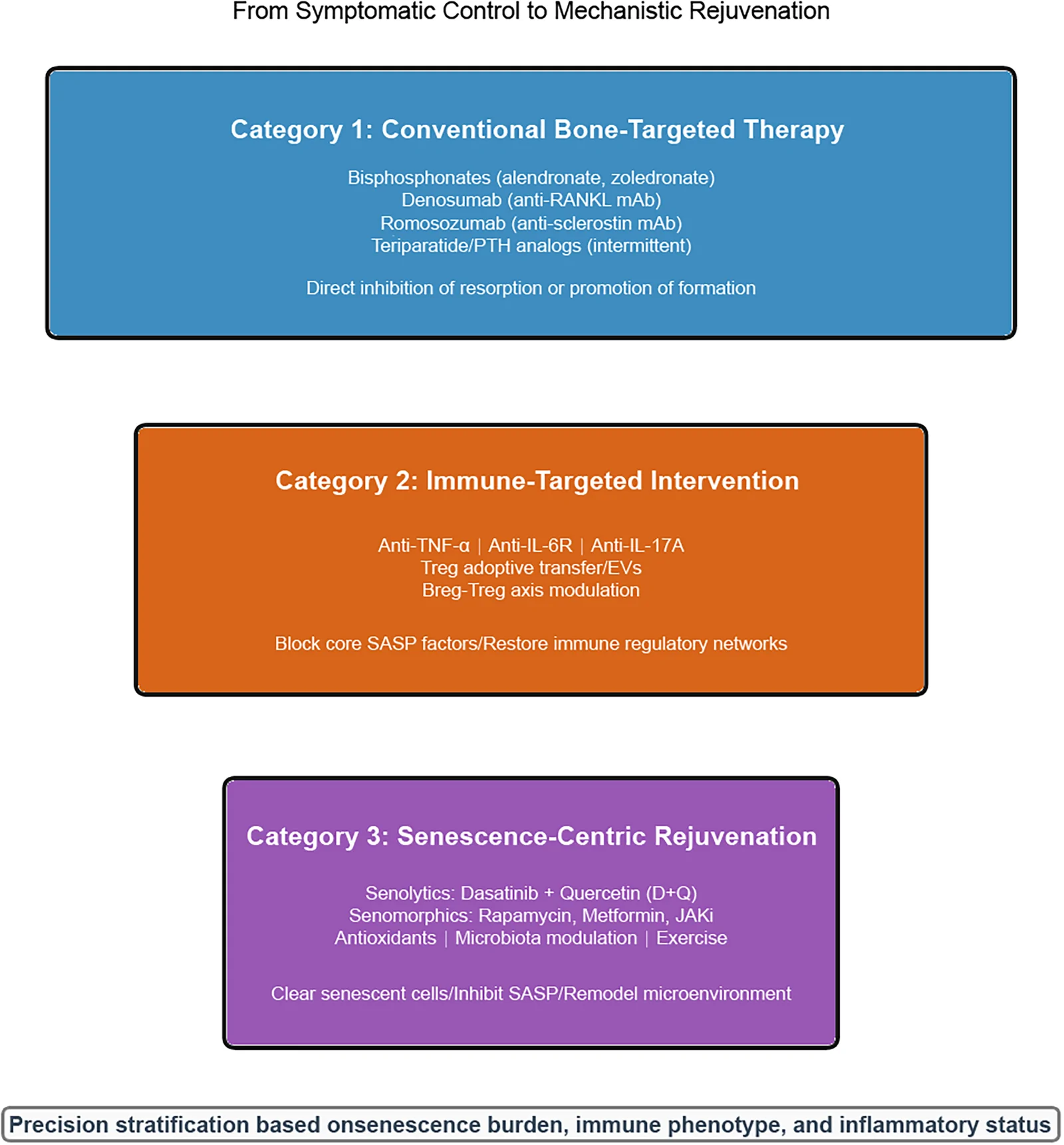
Stratified therapeutic strategies targeting immunosenescence. Treatment approaches are organized into three complementary categories rather than sequential stages: conventional bone-targeted drugs, including bisphosphonates, denosumab, and romosozumab, that directly inhibit bone resorption or promote formation; immune-targeted interventions, such as anti-cytokine antibodies and Treg adoptive transfer, that restore regulatory networks; and senescence-centric strategies, comprising senolytics and senomorphics, that remodel the microenvironment to achieve mechanistic rejuvenation. Adobe Illustrator 2026 software was used to generate the images.

**Table 3:** Classification of anti-bone-loss treatment strategies targeting immunosenescence.

Category	Strategy category	Representative drugs or methods	Main mechanism of action
Category 1	Traditional bone-targeted drugs	Bisphosphonates, denosumab, teriparatide, romosozumab	Approved; directly inhibit bone resorption or promote bone formation, indirectly affect immunity
Category 2a	Inflammatory factor blockade	Anti-TNF-α, anti-IL-6 receptor, anti-IL-17A agents	Approved for inflammatory indications; block core proinflammatory factors of SASP; bone benefit off-label
Category 2b	Immune cell modulation	Treg adoptive transfer, Treg-derived extracellular vesicles, Breg-Treg axis modulation	Investigational; restore immune negative regulatory network
Category 3a	Senescent cell clearance	Dasatinib plus quercetin (D+Q) and other senolytics	Investigational; clear senescent immune cells and bone cells in bone marrow
Category 3b	Senescence phenotype modulation	Rapamycin, metformin, JAK inhibitors, and other senomorphics	Investigational; inhibit SASP production and release
Category 3c	Microenvironment overall regulation	Antioxidant intervention, gut microbiota modulation, inflammation resolution mediators	Preclinical to pilot clinical; reduce systemic inflammaging burden

### 10.1 Traditional bone-targeted drugs

Existing anti-osteoporosis drugs do not directly target immunosenescence, but their actions intersect with the immune system (180, 181). These agents are all approved for osteoporosis, although their indications differ: bisphosphonates and denosumab are indicated for postmenopausal, male, and glucocorticoid-induced osteoporosis, teriparatide for postmenopausal women and for men with osteoporosis at high risk of fracture, and romosozumab for postmenopausal women at high risk of fracture. Bisphosphonates inhibit bone resorption by inducing osteoclast apoptosis and can also be taken up by mononuclear macrophages to produce immunomodulatory effects; denosumab, as an anti-RANKL monoclonal antibody, directly blocks the core signal of osteoclast generation, and RANKL itself has immune regulatory functions. Occasional immune-related adverse events during denosumab treatment, such as autoimmune hepatitis, indirectly confirm the physiological role of the RANKL/RANK axis in the immune system (182). The bone-forming effects of PTH analogs such as teriparatide partially depend on Wnt10b produced by T cells, suggesting that their efficacy may be influenced by the patient’s immune status (165, 181). Understanding the immunological dimension of traditional drugs helps explain individual differences in efficacy and provides a basis for combined medication. Among bone-forming agents, romosozumab, a humanized monoclonal antibody against sclerostin, occupies a distinctive position because it combines anabolic and antiresorptive activities in a single molecule: by neutralizing sclerostin it activates Wnt signaling in osteoblast lineage cells while transiently reducing RANKL-mediated osteoclast activity, which makes it particularly relevant to the coupled formation-resorption imbalance discussed in this review (183, 184). Its relation to osteoimmunology is indirect but notable, because sclerostin is produced mainly by osteocytes and its blockade partially uncouples bone formation from resorption, two processes that immunosenescence drives in opposite directions (183, 184).

The interaction between traditional bone-targeted drugs and the immune system is also reflected in their indirect effects on immune cell function. Bisphosphonates can be sensed by γδT cells and induce their activation; the acute-phase reaction caused by zoledronic acid is an example of immune system participation (181). After denosumab blocks RANKL, in addition to osteoclast inhibition, its effects on RANK signaling in immune cells are also under study (18, 182). Conversely, immune status affects drug response: patients with high inflammatory burden may have different patterns of bone density benefit from anti-resorptive therapy, and the degree of immunosenescence may affect the efficacy of bone-forming drugs (180). These observations have driven attention to the immunopharmacology of bone-targeted drugs (181).

### 10.2 Anti-inflammatory interventions

Biological agents targeting core SASP factors provide direct intervention means for inflammation-driven bone loss. Anti-TNF-α therapy in rheumatoid arthritis and ankylosing spondylitis patients can improve bone density and inhibit local bone erosion; one year of anti-TNF treatment significantly improves patient bone density and bone formation marker levels (185, 186). Comparative studies of denosumab and anti-TNF drugs show that blocking RANKL has more direct effects in inhibiting bone destruction (187). Anti-IL-6 receptor and anti-IL-17A agents also show bone-protective potential in inflammatory arthropathies (117). It should be emphasized that the benefits of inflammatory factor blockade in elderly osteoporosis patients with elevated systemic inflammatory burden still await validation in specifically designed clinical trials (118). For clarity of clinical context, anti-TNF agents, including infliximab, etanercept, and adalimumab, are approved for rheumatoid arthritis, ankylosing spondylitis, and other immune-mediated inflammatory diseases; tocilizumab, an anti-IL-6 receptor antibody, is approved for rheumatoid arthritis and giant cell arteritis; and anti-IL-17A agents, including secukinumab and ixekizumab, are approved for psoriasis, psoriatic arthritis, and ankylosing spondylitis. None of these biologics is currently approved for osteoporosis, and their application to age-related bone loss outside these indications remains investigational.

Evidence for inflammatory factor-targeted interventions also comes from bone metabolism marker studies. After anti-TNF treatment, patient bone resorption markers decrease while bone formation markers are relatively preserved, suggesting that TNF-α blockade breaks the remodeling imbalance under chronic inflammation (185). Clinical studies on the relationship between bone and TNF have established a dose-dependent association between chronic inflammation and bone loss (186). This human evidence supports inflammatory factors as intervenable targets for immunosenescence-related bone loss, but infection risk needs to be balanced, especially in elderly populations (118).

### 10.3 Senolytics and senomorphics

Senolytics and senomorphics represent treatment strategies targeting the aging mechanism itself. The first senolytic combination, dasatinib plus quercetin (D+Q), selectively clears senescent cells by transiently disabling their anti-apoptotic pathways (153, 188). In the skeletal system, clearing senescent cells can prevent age-related bone loss, a concept repeatedly verified in animal models (72, 73). In terms of clinical translation, the Phase II randomized controlled trial by Farr et al. showed that intermittent senolytic treatment had some effect on bone metabolism indicators in postmenopausal women; the decrease in bone resorption markers was more pronounced in the subgroup with higher baseline senescence burden, suggesting that benefit is related to individual senescent cell load (179). D+Q can reduce inflammaging and neutrophil infiltration in aging-related alveolar bone inflammation models (189); at the metabolic level, this combination can also reduce aging-related adipose tissue inflammation and improve metabolic function, suggesting systemic benefits (190). Strategies targeting senescent cells to accelerate fracture healing in the elderly have also shown promise in preclinical studies (93). Senomorphic strategies do not clear senescent cells but inhibit SASP production and release, such as rapamycin, metformin, and JAK inhibitors; their advantage lies in preserving the physiological functions of senescent cells in tissue repair (73, 153). In contrast to the agents discussed in Sections 10.1 and 10.2, no senolytic or senomorphic agent is currently approved for osteoporosis or for any aging indication; intermittent dasatinib plus quercetin remains investigational and has been tested only in early-phase trials (179, 188), while rapamycin and metformin are studied primarily as geroprotective candidates rather than as osteoporosis drugs (44, 46).

The strategic difference between senolytics and senomorphics reflects the dual nature of senescent cell biology. Senescent cells have physiological functions in wound healing, embryonic development, and tumor suppression; complete clearance may bring adverse effects, and the experimental result that local clearance can only partially replicate the benefits of systemic clearance suggests the need for tissue-specific considerations (80). Therefore, intermittent administration to allow tissue recovery, stratified patient selection based on senescent cell burden, and development of tissue-targeted delivery systems are key design principles for clinical application of senolytics (153, 179). In the skeletal field, the bidirectional improvement effects of senescent cell clearance on bone formation and bone resorption have been verified in multiple models, but long-term safety and optimal dosing windows still require large-scale clinical studies (73, 189).

### 10.4 Immune cell therapy and emerging strategies

Active interventions based on immune cells are more forward-looking directions. Local administration of Tregs can promote tissue healing (127); adoptive transfer of bone marrow niche-resident Tregs has shown therapeutic effects in experimental colitis models, suggesting the transplantability of this cell subset (191); IL-10-secreting regulatory B cells can inhibit ovariectomy-induced osteoporosis (192); induced Treg-derived extracellular vesicles inhibit CD4^+^ T cell-mediated inflammation and alleviate bone loss in periodontitis models (128). Immune modulation strategies targeting the Breg-Treg and Th9-Th17 axes have also been proposed under the theoretical framework of postmenopausal osteoporosis (162). Inflammation resolution mediators such as maresin 1 actively promote inflammation resolution to assist bone healing, representing a strategic shift from anti-inflammation to pro-resolution (193). In addition, antioxidant intervention indirectly supports bone formation by protecting MSCs from senescence (152), and gut microbiota modulation affects immunosenescence and bone metabolism at the systemic level (174). Rational combination of these categories may constitute the foundation of future comprehensive management of senile osteoporosis. All of the cell-based approaches and microbiota-based approaches in this category remain preclinical or in early translational stages, and none is approved for bone indications.

## 11 Conclusions and perspectives

The connection between immune cell senescence and bone remodeling imbalance has gained evidentiary support at multiple levels. First, innate immune senescence manifests as macrophage M1/M2 polarization imbalance, increased neutrophil ROS generation and delayed apoptosis, decreased NK cell cytotoxicity and IFN-γ secretion, and impaired DC antigen presentation capacity, jointly creating a proinflammatory, pro-resorptive bone marrow microenvironment (84, 103). Second, adaptive immune senescence manifests as Th17 expansion, Treg functional decline and lineage instability, accumulation of terminally differentiated CD8^+^ T cells, and B cell RANKL/OPG secretion imbalance, directly driving osteoclast activation and inhibiting bone formation (22, 108). Third, senescent immune cells and senescent bone cells mutually amplify each other through SASP, inducing secondary senescence in neighboring cells, causing bone tissue to fall into a vicious cycle of senescence propagation (67, 76). Fourth, systemic hormonal changes such as estrogen deficiency remove constraints on the proinflammatory immune pattern, synergizing with immunosenescence to accelerate bone loss (116, 156).

Several key issues in this field remain to be resolved. Human causal evidence for the effects of senescent immune cells on bone metabolism is still limited; most mechanistic conclusions come from animal models and need to be verified in population cohorts combined with single-cell omics and spatial omics (35). Specific markers and *in vivo* tracing methods for senescent immune cells are not yet well developed, limiting the precision of targeted interventions (64). Clinical studies of senolytics show that benefit is related to baseline senescence burden; how to screen patients most likely to benefit is the key to translation (179). The timing, dosage, and long-term safety of immune interventions, especially in elderly populations with already compromised immune function, require careful evaluation (153). With the proposal of the immunoporosis concept and the deepening of bone immune senescence research, interventions targeting immune cell senescence are expected to become new strategies for delaying skeletal aging, preventing and treating senile osteoporosis, and addressing delayed fracture healing (28, 108, 194).

Future research can be deepened in the following directions. First, establish bone tissue-specific atlases of senescent immune cells. Single-cell and spatial omics technologies have made it possible to resolve the senescence trajectories of bone marrow immune cells; identifying senescence-associated immune subsets specific to the bone microenvironment will provide targets for precision intervention (35, 36). Second, clarify the temporal sequence between senescent immune cells and bone cell senescence. Osteocytes may be the initiating cells of bone tissue senescence, while immune cell senescence exerts remote influence through systemic SASP burden; the causal sequence determines the optimal window for intervention (63, 80). Third, develop bone-targeted intervention delivery strategies for immunosenescence, enabling senolytics and immune modulators to enrich in bone marrow locally while reducing systemic exposure (73). Fourth, promote immune marker-guided stratified management of osteoporosis, incorporating Th17/Treg ratio, inflammatory factor profiles, and senescent cell burden into efficacy prediction systems (108, 179). The progress of research on the interaction between immunosenescence and bone remodeling will drive the management of bone health in the elderly from a simple bone density-oriented approach toward an overall bone immune regulation-oriented approach (28, 194).
